# The wild species genome ancestry of domestic chickens

**DOI:** 10.1186/s12915-020-0738-1

**Published:** 2020-02-12

**Authors:** Raman Akinyanju Lawal, Simon H. Martin, Koen Vanmechelen, Addie Vereijken, Pradeepa Silva, Raed Mahmoud Al-Atiyat, Riyadh Salah Aljumaah, Joram M. Mwacharo, Dong-Dong Wu, Ya-Ping Zhang, Paul M. Hocking, Jacqueline Smith, David Wragg, Olivier Hanotte

**Affiliations:** 1 Cells, Organisms and Molecular Genetics, School of Life Sciences, University of Nottingham, Nottingham, NG7 2RD UK; 2 Present Address: The Jackson Laboratory, 600 Main Street, Bar Harbor, ME 04609 USA; 3 Present Address: Institute of Evolutionary Biology, University of Edinburgh, Edinburgh, EH9 3FL UK; 4 Department of Zoology, University of Cambridge, Cambridge, CB2 3EJ UK; 5 Open University of Diversity - Mouth Foundation, Hasselt, Belgium; 6 Technology and Service B.V., Hendrix Genetics, P.O. Box 114, 5830 AC Boxmeer, The Netherlands; 7 Department of Animal Science, Faculty of Agriculture, University of Peradeniya, Peradeniya, Sri Lanka; 8 Genetics and Biotechnology, Animal Science Department, Agriculture Faculty, Mutah University, Karak, Jordan; 9 Department of Animal Production, King Saud University, Riyadh, Saudi Arabia; 10 Small Ruminant Genomics, International Centre for Agricultural Research in the Dry Areas (ICARDA), P.O. Box 5689, ILRI-Ethiopia Campus, Addis Ababa, Ethiopia; 11 Center for Excellence in Animal Evolution and Genetics, Chinese Academy of Sciences, Kunming, 650223 China; 12 State Key Laboratory of Genetic Resources and Evolution, Kunming Institute of Zoology, Chinese Academy of Sciences, Kunming, 650223 China; 13 The Roslin Institute and Royal (Dick) School of Veterinary Studies, University of Edinburgh, Easter Bush Campus, Midlothian, EH25 9RG UK; 14 Centre for Tropical Livestock Genetics and Health, The Roslin Institute, Edinburgh, EH25 9RG UK; 15 LiveGene, International Livestock Research Institute (ILRI), P. O. 5689, Addis Ababa, Ethiopia

**Keywords:** Chicken introgression, Genetic diversity, Chicken domestication, Livestock species, Divergence time, *Gallus* species, Interspecies hybridisation, Galliformes, Speciation, Evolution

## Abstract

**Background:**

Hybridisation and introgression play key roles in the evolutionary history of animal species. They are commonly observed within several orders in wild birds. The domestic chicken *Gallus gallus domesticus* is the most common livestock species. More than 65 billion chickens are raised annually to produce meat and 80 million metric tons of egg for global human consumption by the commercial sector. Unravelling the origin of its genetic diversity has major application for sustainable breeding improvement programmes.

**Results:**

In this study, we report genome-wide analyses for signatures of introgression between indigenous domestic village chicken and the four wild *Gallus* species. We first assess the genome-wide phylogeny and divergence time across the genus *Gallus*. Genome-wide sequence divergence analysis supports a sister relationship between the Grey junglefowl *G. sonneratii* and Ceylon junglefowl *G. lafayettii*. Both species form a clade that is sister to the Red junglefowl *G. gallus*, with the Green junglefowl *G. varius* the most ancient lineage within the genus. We reveal extensive bidirectional introgression between the Grey junglefowl and the domestic chicken and to a much lesser extent with the Ceylon junglefowl. We identify a single case of Green junglefowl introgression. These introgressed regions include genes with biological functions related to development and immune system.

**Conclusions:**

Our study shows that while the Red junglefowl is the main ancestral species, introgressive hybridisation episodes have impacted the genome and contributed to the diversity of the domestic chicken, although likely at different levels across its geographic range.

## Background

The domestic chicken *Gallus gallus domesticus* plays a key role in human societies. More than 65 billion birds are raised annually to produce meat by the commercial sector [1], and more than 80 million metric tons of egg are produced annually for global human consumption. Despite this importance, the origin and the history of the genetic diversity of this major domesticate are only partly known. The Red junglefowl is the recognised maternal ancestor of domestic chicken [2, 3], with evidence from mitochondrial DNA (mtDNA) supporting multiple domestication centres [4] and the likely maternal contribution of several of its subspecies, with the exception of *G. g. bankiva* (a subspecies with a geographic distribution restricted to Java, Bali, and Sumatra).

**Fig. 1.**
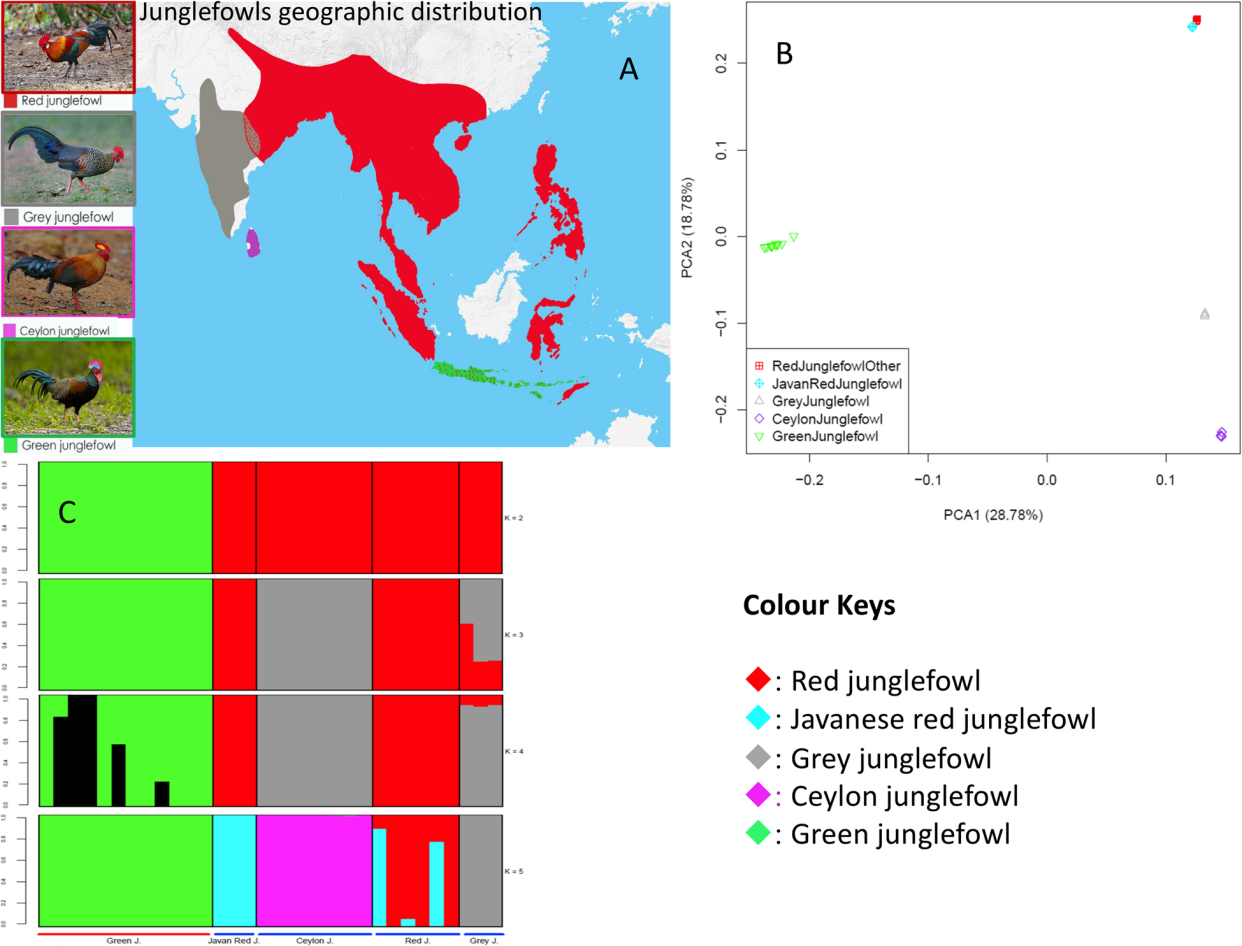
**a** The geographic distribution of the four junglefowl species. The sympatric geographic regions between the Indian red junglefowl (*Gallus gallus murghi*) and the Grey junglefowl on the Indian subcontinent and between the Javanese red junglefowl (*Gallus gallus bankiva*) and the Green junglefowl on the Indonesian Islands are annotated with dots on the map. The map was drawn by overlaying the distribution map of each species obtained from the Handbook of the Birds of the World (consulted in December 2018). Autosomal—**b** principal component and **c** admixture analysis. Junglefowl species photo credits: Peter Ericsson (Red junglefowl), Clement Francis (Grey junglefowl), Markus Lilje (Ceylon junglefowl), and Eric Tan (Green junglefowl)

However, the genus *Gallus* comprises three other wild species, which may have contributed to the genetic background of the domestic chicken. In South Asia, the Grey junglefowl *G. sonneratii* is found in Southwest India and the Ceylon junglefowl *G. lafayettii* in Sri Lanka. In Southeast Asia, the Green junglefowl *G. varius* is endemic to Java and neighbouring islands [5] (Fig. 1a). Hybridisation between the Red and the Grey junglefowls in their sympatric zones on the Indian subcontinent has been documented [5]. In captivity, hybridisation between different *Gallus* species has also been reported [6, 7], with Morejohn successfully producing F1 Red junglefowl × Grey junglefowl fertile hybrids in subsequent backcrossing with both species. Red junglefowl/domestic chicken mtDNA has been found in captive Grey junglefowl [8, 9], and the yellow skin phenotype is likely the result of the introgression of a Grey junglefowl chromosomal fragment into the domestic chicken [10]. Captive F1 hybrids between female domestic chicken and male Green junglefowl, prized for their plumage colour and distinct voice, are common in Indonesia where they are known as Bekisar [5].

More generally, interspecies hybridisation and introgression are an evolutionary processes that play major roles in the genetic history of species and their adaptation [11]. It may occur in the wild when species live in sympatry or in captivity following human intervention. Unravelling how it happens and detecting its signatures at the genome level are central to our understanding of the speciation process. Interspecies hybridisations are commonly practised in agricultural plants and livestock for improving productivity [12], with hybridisation known to occur between domestic and wild species in several taxa [13]. Hybridisation and introgression are also relatively common in wild birds, including in Galliformes [6, 14–17]. For example, the genetic integrity of the rock partridge *Alectoris graeca* is being threatened in its natural habitat through hybridisation with the introduced red-legged partridge *A. rufa* [18], and the presence of Japanese quail alleles in the wild migratory common quail *Coturnix coturnix* reveals hybridisation between domestic quail and the wild relative [19]. Additionally, mtDNA and nuclear microsatellite analyses indicate gene flow between the Silver Pheasant *Lophura nycthemera* and Kalij Pheasant *L. leucomelanos* [20]. Infertile F1 hybrids between the common Pheasant *Phasianus colchicus* and domestic chicken have also been reported in captivity [21].

Here, we report whole-genome analysis of indigenous domestic village chickens from Ethiopia, Saudi Arabia, and Sri Lanka, together with domestic breeds from Indonesia and China, European fancy chickens, and the four wild junglefowl species to infer the genetic contributions of different *Gallus* species to the domestic chicken genome. We first assess the phylogeny of the genus. It supports (i) a sister relationship between the Grey junglefowl and the Ceylon junglefowl with the clades of both species being sister to the Red junglefowl, (ii) the Green junglefowl as the most ancient lineage within the genus, and (iii) that the domestication of the chicken from the Red junglefowl occurred around 8000 years ago. We then show introgression in domestic chicken from the three non-red junglefowl species (Grey, Ceylon, and Green). We also observe extensive introgression from the domestic chicken/Red junglefowl into the Grey junglefowl and some introgression from the domestic chicken into Ceylon junglefowl. Our findings indicate that the genome diversity of domestic chicken, while originating from the Red junglefowl, was subsequently reshaped and enhanced following introgression from other *Gallus* species, although with different impact according to the history of each domestic chicken population.

## Results

### Sampling, genetic structure, and diversity

We analysed 87 whole-genome sequences from domestic chickens (*n* = 53), Red junglefowl (Red (*n* = 6) and Javanese red (*n* = 3)), Grey junglefowl (*n* = 3), Ceylon junglefowl (*n* = 8), and Green junglefowl (*n* = 12) and common Pheasant (*n* = 2). Our dataset comprised newly sequenced genomes at an average depth of 30×, together with publicly available sequence data, which ranged from 8× to 14×. Across all the 87 genomes, 91,053,192 autosomal single nucleotide polymorphisms (SNPs) were called. Summary statistics for read mapping and SNPs are provided in Additional file 1: Table S1.

To understand the genetic structure and diversity of the four *Gallus* species, we ran principal component (PC) and admixture analyses based on the autosomal SNPs filtered to control for linkage disequilibrium. PC1 clearly separates the Green junglefowl from the other *Gallus* species, while PC2 separates the Red, Grey, and Ceylon junglefowls (Fig. 1b), with the Grey and Ceylon junglefowls positioned closer to each other compared to the Red and Green junglefowls. PC2 also separates the Javanese red junglefowl subspecies from the other Red junglefowls. The admixture analysis recapitulates these findings, providing some evidence for shared ancestry between the Red and Grey junglefowls at *K* = 3, but at the optimal *K* = 5, the ancestry of each junglefowl species is distinct (Fig. 1c).

### Detecting the true *Gallus* species phylogeny

**Fig. 2.**
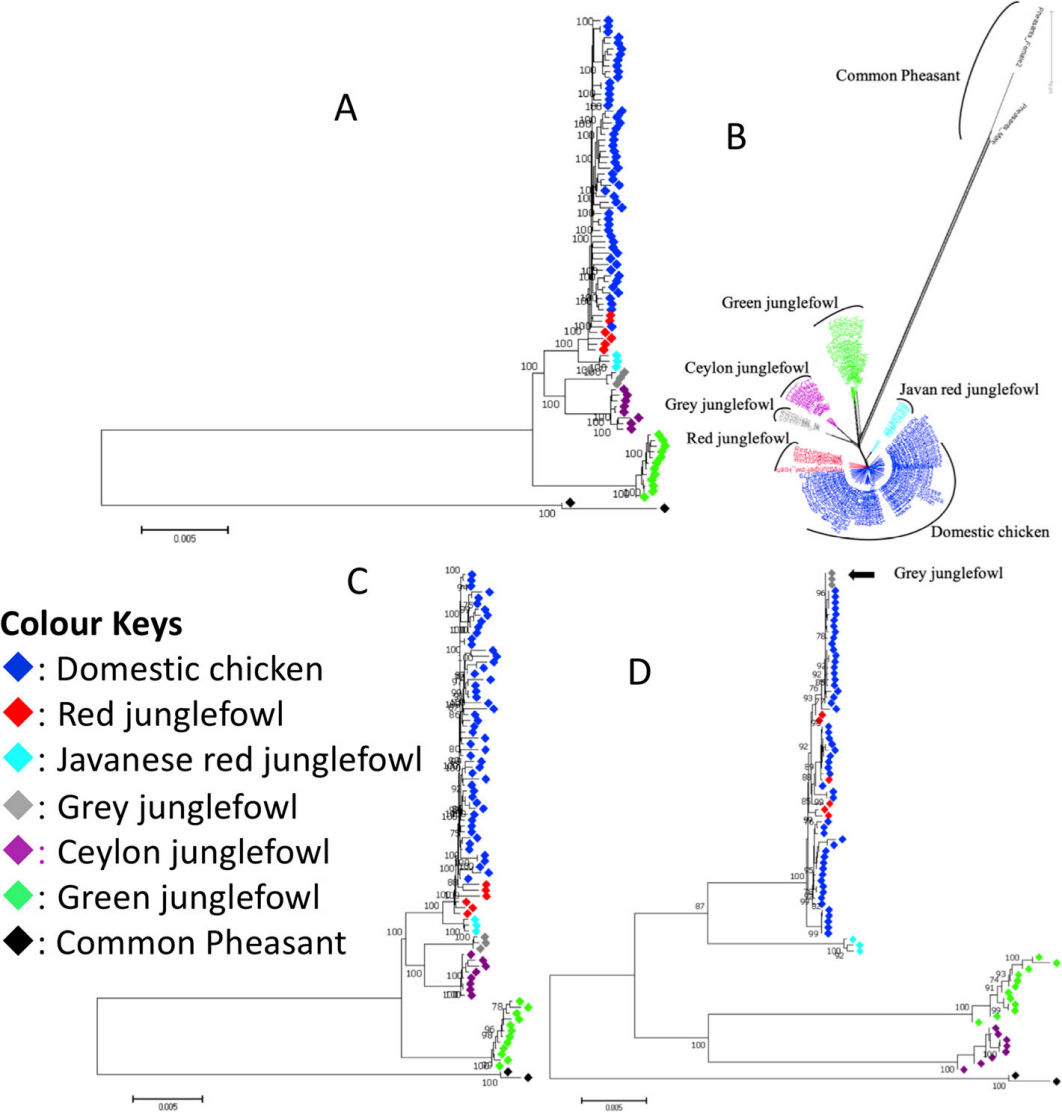
The genome-wide phylogeny of the genus *Gallus.* **a**, **c**, **d** Neighbour-joining phylogenetic trees for the autosomes, Z chromosome, and mitochondrial DNA, respectively. **b** The distance matrix of the autosomes constructed from the NeighborNet network of SplitsTree4. **d** The three Grey junglefowl mtDNA haplotypes embedded within the domestic/Red junglefowl lineage are indicated with a black arrow. All the trees are rooted with the common Pheasant *Phasianus colchicus*

We constructed a neighbour-joining tree and a NeighborNet network using autosomal sequences of 860,377 SNPs separated by at least 1 kb from a total of 91 Million SNPs, and a maximum likelihood tree on 1,849,580 exon SNPs extracted from the entire autosomal whole-genome SNPs. The trees were rooted with the common Pheasant as the outgroup (Fig. 2a, b; Additional file 2: Figure S1A). Our results show that the Grey and the Ceylon junglefowls are sister species and form a clade that is sister to the clade of the Javanese red junglefowl, the Red junglefowl, and the domestic chicken, with the latter two being paraphyletic. The Green junglefowl is outside of this clade, making it the most divergent junglefowl species. We also observe the same relationships for the Z chromosome as well as for the mitochondrial (mt) genome (Fig. 2c, d, respectively). However, the latter shows that the studied Grey junglefowl do carry a domestic/Red junglefowl mitochondrial haplotype. All the trees show the Javanese red junglefowl lineage at the base of the domestic/Red junglefowl lineages.

Next, we investigated the extent to which other topologies are represented in the autosomal genome using topology weighting by the iterative sampling, based on windows of 50 SNPs, of sub-trees (*Twisst*) [22]. First, we estimate the admixture proportion for the autosomal genome shared between domestic chicken and Red junglefowl. We obtain 71% for *Twisst* estimation based on the sum of topologies T1–T3, which show a monophyletic relationship between the domestic chicken and Red junglefowl (Additional file 2: Figure S1C).

The analysis was then performed thrice using either the domestic chicken, the Red junglefowl, or the Javanese red junglefowl along with the Grey, Ceylon, and Green junglefowls and the common Pheasant (outgroup). *Twisst* estimates the relative frequency of occurrence (i.e. the weighting) of each of the 15 possible topologies for these 5 taxa for each window and across the genome.

**Fig. 3.**
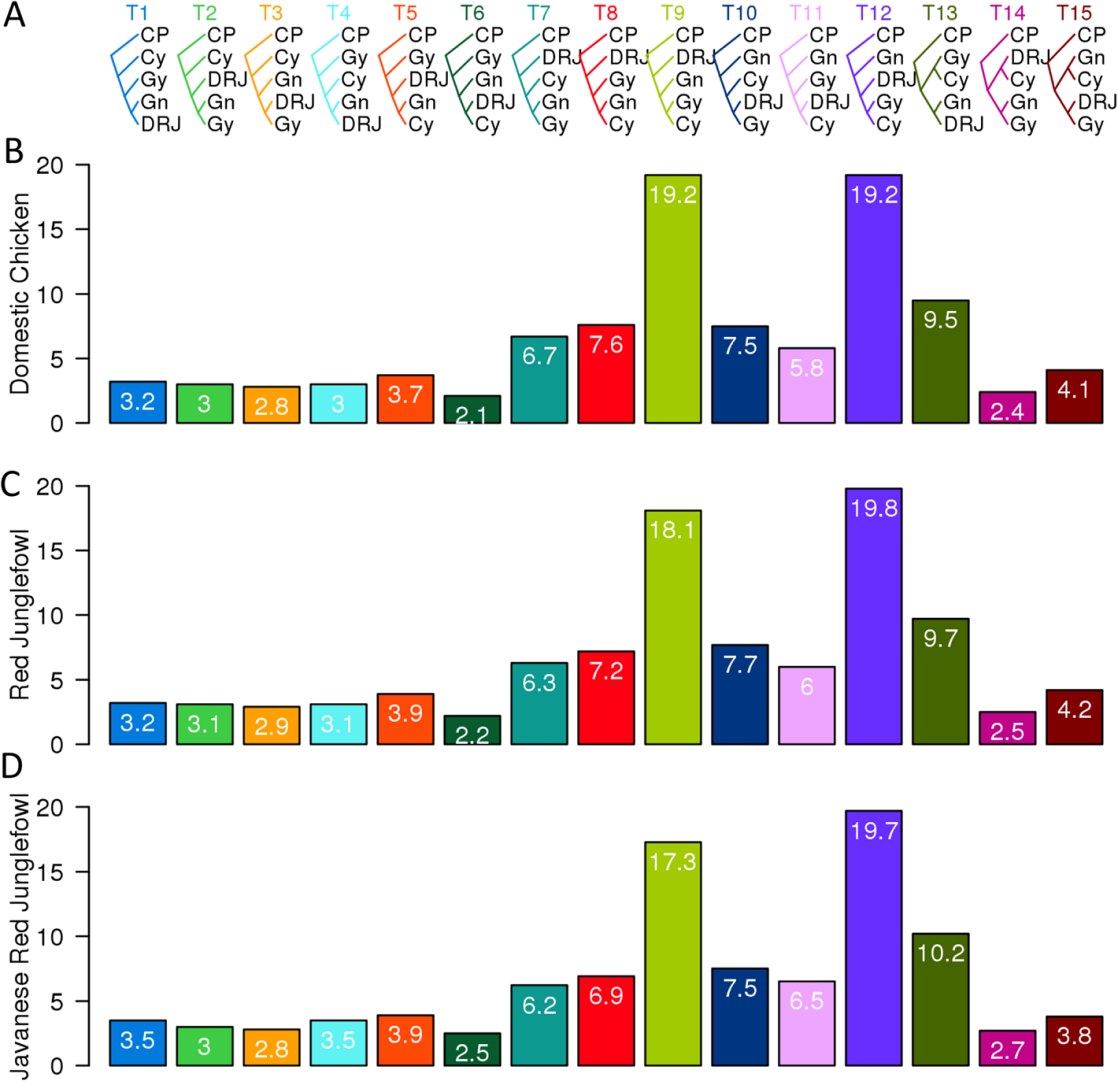
Topology weighting by iterative sampling of sub-trees (*Twisst*). **a** The 15 possible topologies (T1–T15) from 5 taxa. As the number of possible topologies works best for a maximum of 5 taxa [22] and with the presence of 7 taxa in this study, we ran the analysis thrice: with **b** domestic chicken “D,” **c** Red junglefowl “R,” and **d** Javanese Red junglefowl “J.” The average weightings (%) for each of the 15 topologies are included in each bar and as well as indicated on the *Y* axis. Domestic chicken or Red junglefowl or Javanese red junglefowl (DRJ), Grey junglefowl (Gy), Ceylon junglefowl (Cy), Green junglefowl (Gn), and common Pheasant (CP)

The most highly weighted topology genome-wide (T12), accounting for ~ 20% of the genome, supports the autosomal species genome phylogeny: ((((Domestic chicken or Red junglefowl or Javanese red junglefowl), (Grey junglefowl, Ceylon junglefowl)), Green junglefowl), common Pheasant) (Fig. 3), while the second-highest topology, T9 (ranges 18–19%), instead places the Green junglefowl as sister species to the Grey and Ceylon junglefowls: ((((Grey junglefowl, Ceylon junglefowl), Green junglefowl), Domestic or Red junglefowl or Javanese red junglefowl), common Pheasant). There are also weightings for other topologies. In particular, topologies 3 (~ 2.9%), 10 (~ 7.7%), and 15 (~ 4.2%) show sister relationships between the Domestic/Red junglefowl and the Grey junglefowl; topologies 6 (~ 2.2%) and 11 (~ 6%) between the Ceylon junglefowl and the Domestic/Red junglefowl; and topologies 1 (~ 3.2%), 4 (~ 3.1%), and 13 (~ 9.7%) between the Green junglefowl and the Domestic/Red junglefowl.

The result of TreeMix shows similar trends in phylogenetic relationships (as above), but it indicates multiple histories of admixture, namely from the Red junglefowl to the Grey junglefowl, from the Ceylon junglefowl to the Red junglefowl, and from the root of the monophyly Grey and Ceylon junglefowls to the Green junglefowl (Additional file 2: Figure S1B), with the latter being consistent with topology 9 in Fig. 3a.

### Species divergence time

We used two approaches for the estimation of divergence time between lineages. We first measured the autosomal average absolute pairwise sequence divergence between each species pair. This measure represents the sum of accumulated sequence divergence since speciation and pairwise nucleotide differences existed in the ancestral population. To estimate the species split time, we adjusted this measure of divergence downward by subtracting an estimated ancestral diversity, which we took as the average diversity between two taxa (i.e. *d*_*a*_ [23]). Times are reported in years (see the “Materials and methods” section). Among the junglefowls, the divergence times span a few million years, namely, ~ 1.2 MYA (Million Years Ago) between the Red and Javanese red junglefowls, ~ 1.8 MYA between the Grey and Ceylon junglefowls, ~ 2.6 to 2.9 MYA between the Red/Javanese red and Grey/Ceylon junglefowls, and ~ 4 MYA between the Green and the other junglefowl species, while the junglefowl species and the common Pheasant lineages diverged ~ 21 MYA (see Table 1 for details of all the pairwise divergence calculations). These split times agree with the autosomal and Z chromosome species tree relationships (Fig. 2). Using the same approach, we estimate 8093 (CI 7014–8768) years for the accumulated divergence time (domestication) between the domestic chicken and Red junglefowl (Table 1).

**Table 1:** Divergence time (direct estimates) between junglefowl species and with the common Pheasant

Pairwise species comparison	Divergence time (DT) in years*	95% confidence interval (years)
Domestic chicken–Red junglefowl	8093	7014 ≤ DT ≤ 8768
Red junglefowl–Javanese red junglefowl	1,164,612	1,009,331 ≤ DT ≤ 1,261,663
Red junglefowl–Grey junglefowl	2,557,021	2,216,085 ≤ DT ≤ 2,770,106
Javanese red junglefowl–Grey junglefowl	2,646,356	2,293,509 ≤ DT ≤ 2,866,886
Grey junglefowl–Ceylon junglefowl	1,766,945	1,531,352 ≤ DT ≤ 1,914,191
Red junglefowl–Ceylon junglefowl	2,842,140	2,463,188 ≤ DT ≤ 3,078,985
Javanese red junglefowl–Ceylon junglefowl	2,864,596	2,482,650 ≤ DT ≤ 3,103,312
Red junglefowl–Green junglefowl	4,057,810	3,516,769 ≤ DT ≤ 4,395,961
Javanese red junglefowl–Green junglefowl	4,059,609	3,518,328 ≤ DT ≤ 4,397,910
Grey junglefowl–Green junglefowl	3,992,696	3,460,337 ≤ DT ≤ 4,325,421
Ceylon junglefowl–Green junglefowl	3,997,328	3,464,351 ≤ DT ≤ 4,330,438
Red junglefowl–Common Pheasant	20,736,660	17,971,772 ≤ DT ≤ 22,464,715
Javanese red junglefowl–Common Pheasant	20,934,414	18,143,159 ≤ DT ≤ 22,678,949
Grey junglefowl–Common Pheasant	20,986,911	18,188,656 ≤ DT ≤ 22,735,820
Ceylon junglefowl–Common Pheasant	21,025,261	18,221,892 ≤ DT ≤ 22,777,366
Green junglefowl–Common Pheasant	21,361,699	18,513,472 ≤ DT ≤ 23,141,840

*Assuming one generation per year

We then compared the direct estimate result with ∂a∂i which uses a model-based inference approach on joint site frequency spectrum (SFS) that takes into consideration the effective population sizes and migration between species. We estimated ∂a∂i from SFS using the entire genome information obtained from the binary alignment map files. On average and across the different pairwise analyses, our results indicate that the ancestor of the genus *Gallus* had an effective population size of at least 1 million. As ∂a∂i uses the SFS, pairwise divergence times with Grey junglefowl, Javanese red junglefowl, and common Pheasant were not included in this analysis due to small sample sizes. The divergence times were estimated as ~ 5.7 MYA (CI 4.9–6.1 MYA) between the Red and the Green junglefowls, ~ 3.0 MYA (CI 2.6–3.2 MYA) between the Red and the Ceylon junglefowls, ~ 2.2 MYA (CI 1.9–2.4 MYA) between the Ceylon and Green junglefowls, and 81 KYA (70–89 KYA) between domestic chicken and Red junglefowl (Table 2).

**Table 2:** ∂a∂i divergence time estimates between junglefowl species

Pairwise species comparison	Divergence time (DT) in years*	95% confidence interval (years)
Domestic chicken–Red junglefowl	81,215	70,386 ≤ DT ≤ 87,983
Red junglefowl–Ceylon junglefowl	2,963,109	2,568,028 ≤ DT ≤ 3,210,035
Red junglefowl–Green junglefowl	5,659,029	4,904,492 ≤ DT ≤ 6,130,615
Ceylon junglefowl–Green junglefowl	2,181,977	1,891,046 ≤ DT ≤ 2,363,808

*Assuming one generation per year

### Genome-wide tests for introgression between junglefowl and domestic chicken

Having established general patterns for the evolutionary history and relationships among the junglefowl species, we next assess the presence of shared alleles between the domestic chicken and the *Gallus* species. We used *D*-statistics [24, 25] to test for a genome-wide excess of shared alleles between the domestic chicken and each of the non-red junglefowl species, relative to the Red junglefowl. *D* is significantly greater than zero with strong *Z*-scores in all three cases (Table 3), implying possible introgression between domestic chicken and the Grey, Ceylon, and Green junglefowls. However, because the Grey and the Ceylon junglefowls are sister species, introgression from just one of these species into domestic chicken could produce significantly positive *D* values in both tests. Accordingly, the estimated admixture proportions (*f*) are similar in both cases, ~ 12% and ~ 14% for the Grey and the Ceylon junglefowls, respectively. The estimated admixture proportions are lower for the Z chromosomes, ~ 6% with the Grey junglefowl and ~ 10% with the Ceylon junglefowl. Between the domestic chicken and the Green junglefowl, they are ~ 9% for the autosomes and ~ 7% for the Z chromosome.

**Table 3:** Patterson’s *D*-statistics and admixture proportion

Domestic	Junglefowl	Patterson’s *D*-statistics			Admixture proportion (*f*)	
		*D*	Jackknife SD	*Z*-score	*f* estimates	95% confidence interval
Autosomes (chromosomes 1–28)						
Domestic	Grey junglefowl	0.07	0.06	37.85	0.12	0.11 ≤ *f* ≤ 0.14
Domestic	Ceylon junglefowl	0.06	0.05	36.78	0.14	0.13 ≤ *f* ≤ 0.10
Domestic	Green junglefowl	0.05	0.05	34.24	0.09	0.08 ≤ *f* ≤ 0.09
Z chromosome						
Domestic	Grey junglefowl	0.04	0.09	4.18	0.06	0.03 ≤ *f* ≤ 0.09
Domestic	Ceylon junglefowl	0.04	0.09	4.51	0.10	0.06 ≤ *f* ≤ 0.14
Domestic	Green junglefowl	0.04	0.09	4.25	0.07	0.04 ≤ *f* ≤ 0.10

We also estimated the admixture proportion (*f*) for the autosomal genome between the domestic chicken and the Red junglefowl. We obtained a value of 79% between the two species, which is closer to the 71% from the *Twisst* tree proportion estimation (Additional file 2: Figure S1C).

### Genome scans for introgressed regions

**Fig. 4.**
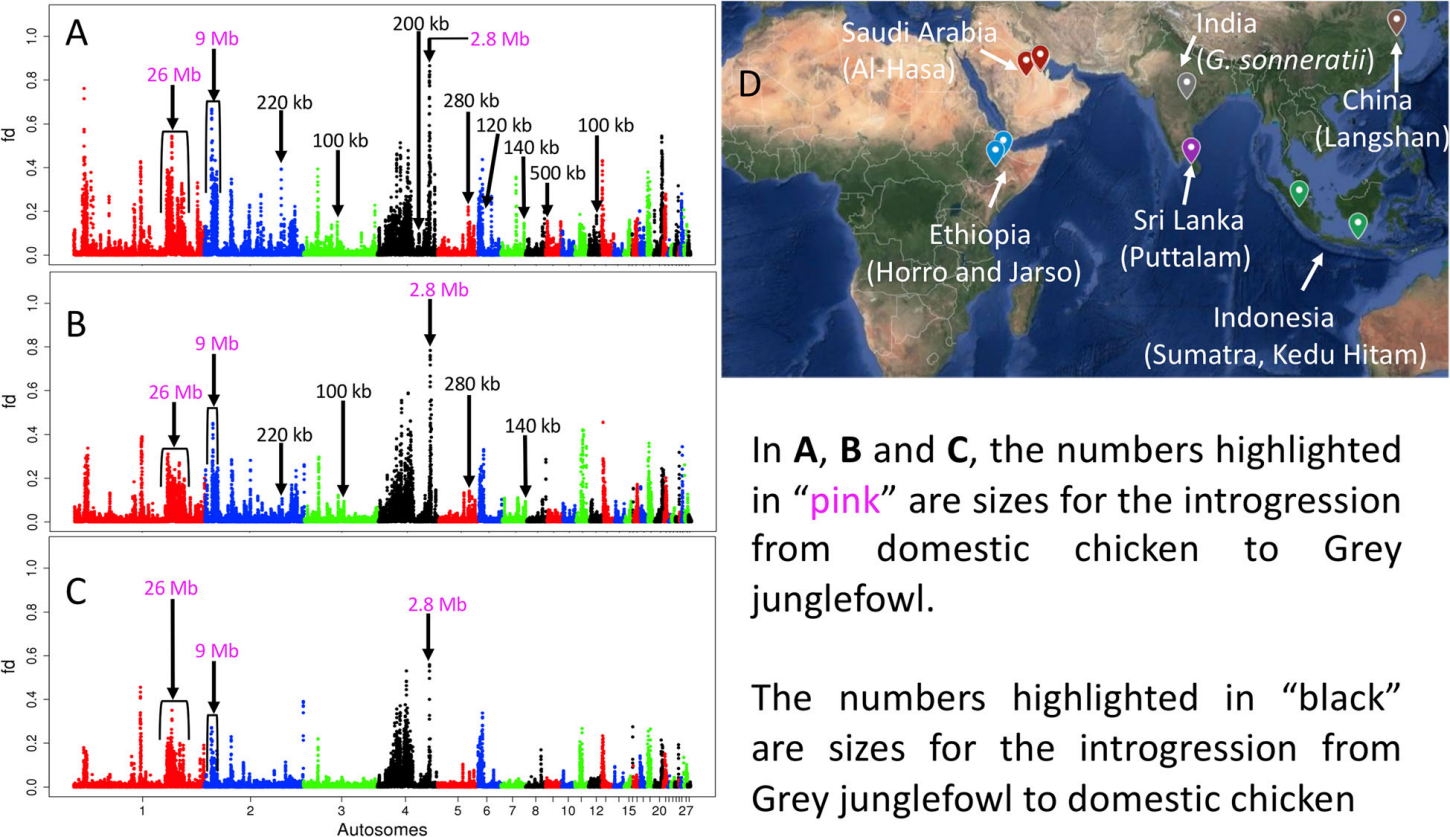
The *f*_*d*_ plots test for the comparison between the Grey junglefowl and the domestic chicken populations from **a** Ethiopia and Saudi Arabia, **b** Sri Lanka, and **c** Southeast Asia (Indonesia), and East Asia (China). **d** Geographical map showing the countries and regions of origin for each domestic chicken population. The Grey junglefowl *G. sonneratii* geographic distribution is India*.* Genes within the candidate regions highlighted by their sizes are described in Additional file 4: Table S2 and Additional file 8: Table S3. *Y* axis: *f*_*d*_ value spanning 0 to 1, *X* axis: autosomal chromosomes number from 1 to 28. See Additional files 16 and 19 for the domestic–Ceylon and the domestic–Green junglefowl comparisons, respectively

To identify specific loci harbouring introgressed allele, we calculated *f*_*d*_ [26], which estimate local admixture proportion within a defined 100 kb window size. This window size was chosen because it is much greater than the expected size of tracts of shared ancestry from incomplete lineage sorting (ILS) between these species. Given their estimated divergence time and a recombination rate of 3 × 10^−8^, tracts of shared variation across the species that resulted from ILS would be expected to be very small, at the order of ~ 8 bp (95% CI 7–10 bp) on average (see the “Materials and methods” section). Next, we separated the domestic chicken into three groups based on their geographic origin and in relation to the geographic location of the junglefowl species: (*i*) Ethiopian and Saudi Arabian domestic chickens (West of the Grey and wild Red junglefowl geographic distribution), (*ii*) Sri Lankan domestic chicken inhabiting the same island as the Ceylon junglefowl, and (*iii*) Southeast and East Asian domestic chickens, which include two breeds (Kedu Hitam and Sumatra) from the Indonesian Islands, a geographic area where the Red and the Green junglefowls are found, and the Langshan, a breed sampled in the UK but originally from China (Fig. 1a and Fig. 4d).

Candidate introgressed loci revealed by *f*_*d*_ are further supported by additional statistics including the relationship among topologies and proportion of admixture at the introgressed locus, nucleotide divergence (*dxy*), genetic differentiation (*Fst*), and haplotype network/tree. We tested these approaches first on the well-established yellow skin introgressed locus in chicken (chr24: 6,107,101–6,135,115 bp, based on GRCg6a reference). The results from these statistics are consistent with prior published results [10] for yellow skin in domestic chicken, which are grouped with the Grey junglefowl. The non-yellow skin carriers are grouped with the Red junglefowl (Additional file 3: Figure S2).

For the introgression analysis between the domestic chicken and Grey junglefowl, most of the peaks are introgressed from the domestic/Red junglefowl into Grey junglefowl (see Raman Akinyanju Lawal PhD thesis [9]). We selected here the three most extreme *f*_*d*_ peaks that are consistent across all three domestic chicken groups for further investigation (Fig. 4): a 26-Mb region on chromosome 1 at chromosomal position 141,287,737–167,334,186 bp, a 9-Mb region on chromosome 2 at position 11,022,874–19,972,089 bp, and a 2.8-Mb region on chromosome 4 at position 76,429,662–79,206,200 bp (Additional file 4: Table S2; Fig. 6a; Additional file 5: Figure S3A, Additional file 6: Figure S4A, Additional file 7: Figure S5A). Both the haplotype trees and networks show nesting of some Grey junglefowl haplotypes within the domestic chicken lineage, consistent with introgression from the domestic chicken/Red junglefowl into the Grey junglefowl (Additional file 5: Figure S3, Additional file 6: Figure S4, Additional file 7: Figure S5 (B–C)). These results are further supported by *Twisst*, which indicates localised reductions in the weighting of the species topology and increases in the weightings for both the topologies (((Grey junglefowl, domestic), Red junglefowl), common Pheasant) and (((Grey junglefowl, Red junglefowl), domestic), common Pheasant) (Additional file 5: Figure S3D, Additional file 6: Figure S4D, Additional file 7: Figure S5D). Furthermore, at the candidate introgressed region, *dxy* and *Fst* are reduced between domestic chicken and Grey junglefowl, but not between domestic chicken and Red junglefowl (Additional file 5: Figure S3, Additional file 6: Figure S4, Additional file 7: Figure S5 (E–F)). These large genomic regions show all the signals expected of recent introgression from the domestic chicken/Red junglefowl into the Grey junglefowl.

**Fig. 5.**
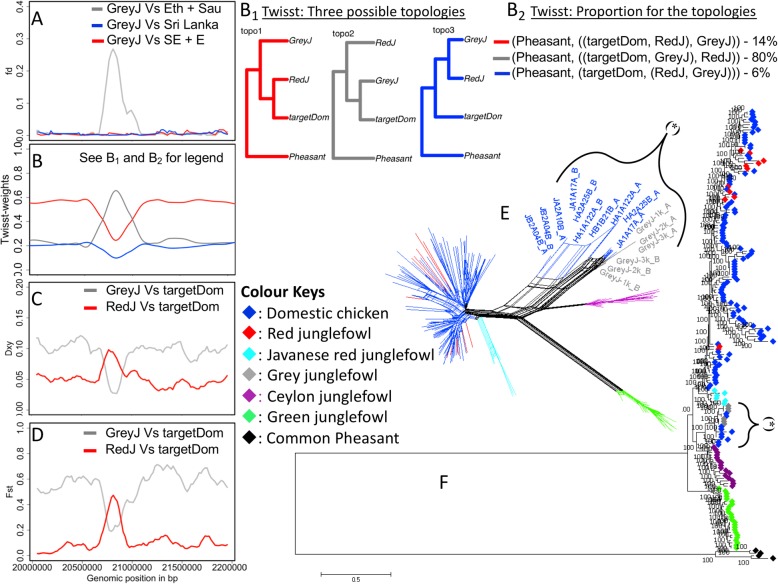
A 120-kb (Chr 6: 21,729,370–21,849,500 bp, based on GRCg6a reference) introgressed region from the Grey junglefowl into the domestic chicken. **A** *f*_*d*_ plot. **B** *Twisst* plot (**B**_**1**_ its topologies and **B**_**2**_ their proportions). The most consistent topology (80%) has a monophyletic relationship between targetDom (introgressed domestic haplotypes) and Grey junglefowl. **C** *dxy* and **D** *Fst*. Eth, Sau, SriLanka, and SE + E are domestic chickens from Ethiopia, Saudi Arabia, Sri Lanka, and Southeast Asia (Indonesia) + East Asia (China), respectively. targetDom are the introgressed domestic chicken haplotypes from Grey junglefowl (GreyJ) denoted as (*) in **E** haplotype-based network and **F** maximum likelihood tree

Next, we investigated inconsistent candidate introgression across the three domestic chicken geographic group comparisons, i.e. peaks present only in one or two comparisons. Fig. 4a clearly represent most of these introgression signals. We then selected eight peaks (Additional file 8: Table S3). The sequence length for these regions ranges from 100 to 500 kb. Haplotype trees and networks show that domestic chicken haplotypes (referred to here as targetDom) are nested within or close to the Grey junglefowl ones, supporting introgression from Grey junglefowl into domestic chicken at these regions (Fig. 5A; Additional file 9: Figure S6, Additional file 10: Figure S7, Additional file 11: Figure S8, Additional file 12: Figure S9, Additional file 13: Figure S10, Additional file 14: Figure S11, Additional file 15: Figure S12). *Twisst* results indicate localised increases in the weighting for the topology (((Grey junglefowl, targetDom), Red Junglefowl), common Pheasant) with proportions ranging from 61 to 80%, much higher than the species topology (((Red junglefowl, targetDom), Grey junglefowl), common Pheasant) ranging from 14 to 28%, and the other alternative topology (((Grey junglefowl, Red junglefowl), targetDom), common Pheasant) ranging from 6 to 11%. These loci are also characterised by reduced *dxy* and *Fst* values between the Grey junglefowl and the domestic chicken and by increased *dxy* and *Fst* between the Red junglefowl and the domestic chicken (Fig. 5; Additional file 9: Figure S6, Additional file 10: Figure S7, Additional file 11: Figure S8, Additional file 12: Figure S9, Additional file 13: Figure S10, Additional file 14: Figure S11, Additional file 15: Figure S12 (E–F)). These Grey junglefowl introgressed regions are mainly found in the Ethiopian chickens (*n* = 8) than in the Saudi Arabian chicken (*n* = 3). Four regions are also found in Sri Lankan chicken, two in Sumatran chicken, one each in Kedu Hitam chicken, and one in wild Red junglefowl (Additional file 8: Table S3). The introgression found on chromosome 5 was also present in European fancy chicken breed (Mechelse Koekoek, Additional file 12: Figure S9). No Grey junglefowl introgression is detected in the Langshan chicken. Across these eight regions, a 100-kb candidate for bidirectional introgression is observed on chromosome 12 with a single Grey junglefowl haplotype nested within the domestic/Red junglefowl lineage (Additional file 15: Figure S12).

**Fig. 6.**
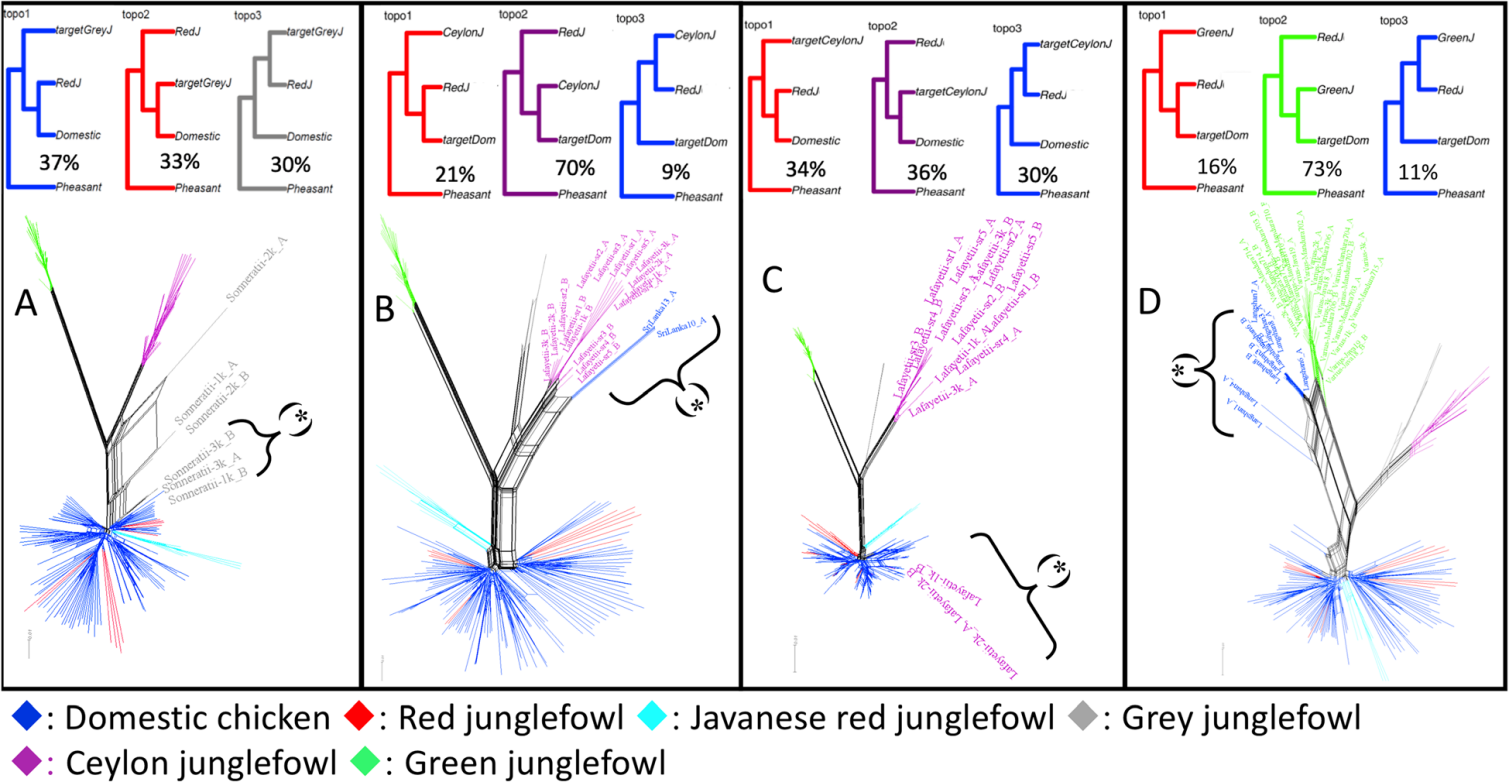
Topologies (*Twisst*), their estimated proportions, and network analyses for the introgression from **a** domestic chicken to Grey junglefowl (2.8 Mb, Chr 4: 76,429,662–79,206,200 bp), **b** Ceylon junglefowl to domestic chicken (600 kb, Chr 3: 108,325,801–108,925,700 bp), **c** domestic chicken/Red junglefowl to Ceylon junglefowl (100 kb, Chr 5: 49,333,700–49,433,700 bp), and **d** Green junglefowl to domestic chicken (100 kb, Chr 5: 9,538,700–9,638,700 bp), based on GRCg6a reference. (*) introgressed haplotypes. The targetGreyJ, targetDom, and targetCeylon in the *Twisst* are the introgressed, as revealed by the network, Grey junglefowl, domestic chicken, and Ceylon junglefowl haplotypes, respectively

A smaller number of candidate regions are detectable in *f*_*d*_ between domestic chicken and Ceylon junglefowl (Additional file 16: Figure S13). In most of the candidate regions investigated, haplotype trees and networks indicate unresolved relationships, whereas some show introgression from Grey rather than Ceylon junglefowl into the domestic chicken. By further analysing every peak in the plot, we identified four candidate introgressed regions from Ceylon junglefowl into the domestic chicken: three on chromosome 1, spanning 6.52 Mb, 3.95 Mb, and 1.38 Mb; and one on chromosome 3, spanning 600 kb (Additional file 8: Table S3). The haplotype networks and other statistics show introgression of Ceylon junglefowl into a single haplotype of domestic chicken from Sri Lanka for the three candidate regions on chromosome 1 (Additional file 17: Figure S14), and into two Sri Lankan domestic chickens for the chromosome 3 region (Fig. 6b; Additional file 18: Figure S15). The 1.38-Mb region on chromosome 1 also shows introgression from domestic/Red junglefowl into Grey junglefowl (Additional file 17: Fig. S14C). For the four introgressed regions, *Twisst* shows the highest weighting for a topology grouping the target domestic chicken samples with Ceylon junglefowl. Only one candidate region, a 100-kb region, on chromosome 5 shows evidence of introgression from domestic/Red junglefowl into Ceylon junglefowl. This introgression is supported by both the haplotype network and the topology weightings (Additional file 4: Table S2; Fig. 6c).

There are several peaks of elevated *f*_*d*_ between Green junglefowl and the domestic chicken groups (Additional file 19: Figure S16). However, both the haplotype tree and network support introgression only in a single case, at a 100-kb region on chromosome 5 at position 9,538,700–9,638,700 bp (Fig. 6d; Additional file 20: Figure S17). Here, the introgression was present in 10 out of 16 Langshan haplotypes (Additional file 8: Table S3). This introgression was supported by high weighting for the topology grouping the introgressed domestic chicken samples with the Green junglefowl, as well as reduced *d*_*XY*_ and *F*_*ST*_ between domestic chicken and Green junglefowl (Additional file 20: Figure S17).

## Discussion

The Red junglefowl has long been known as the ancestor of domestic chicken [2–4]. However, one molecular study has shown the presence of an autosomal DNA fragment from the Grey junglefowl in the genome of some domestic chicken [10], whereas other studies revealed the presence of Red junglefowl/domestic chicken mitochondrial DNA in the Grey junglefowl [8, 9]. Also, F1 crossbreeding of domestic birds with the Green junglefowl is common [5] and captive breeding experiments have reported, although, at a very low rate, hatching of eggs and survival of chicks from F1 female Grey × Red junglefowl birds backcrossed to male parental birds from each species [6, 7]. These studies suggest that other species within the genus *Gallus* may have contributed to the diversity of the domestic chicken gene pool. Here, we report for the first time an analysis of the full genomes of the four wild junglefowl species to assess their level of contribution to the diversity of the domestic chicken genomes.

We first established the species phylogeny using genome sequence comparison of the genus *Gallus.* The phylogenies constructed from the autosomes and Z chromosome placed the Red/Javanese red junglefowl equally close to the Grey and the Ceylon junglefowls, which show a sister species relationship. Both also indicate that the Green junglefowl lineage was the first to separate from the common ancestry of the genus. Interestingly, the separation of the Javanese red junglefowl, around 1.2 MYA, occurs at the root of other Red junglefowl samples studied here, noting that the latter did not include any representative of the Red junglefowl subspecies *G. gallus murghi* from the Indian subcontinent. The *Gallus* phylogeny supports a Southeast Asian origin for the genus, with a first lineage splitting event separating the Green junglefowl on the present-day Indonesian Islands ~ 4–6 MYA, at the time boundary between the Pliocene and early Pleistocene. Then, a North and Northwest dispersion of the Red junglefowl ancestral population led to the separation, possibly on the Indian subcontinent, of the lineages leading to the Grey and the Ceylon junglefowls ~ 2.6 to 2.9 MYA. It was followed by the speciation of the Grey and the Ceylon junglefowls ~ 1.8 MYA. Using the same approach, we estimated that the domestication of chicken from Red junglefowl likely occurred ~ 8000 years ago (95% CI 7014–8768 years), around 2000–3000 years earlier than the archaeological evidence on the North of the Indian subcontinent [27] and China [28], but within the Neolithic period.

The divergence time between the Ceylon and the Red junglefowls as well as between the Green and the Red junglefowls is similar for the absolute pairwise sequence divergence estimation and the model-based ∂a∂i approach. However, it is not the case for the divergence time between the Ceylon and the Green junglefowls. This result is surprising considering the autosomal, Z chromosome, and mitochondrial tree relationships of the genus. However, topology weighting analysis shows considerable discordance in relationships across the genome, with weightings for topologies grouping Red junglefowl/domestic chicken with other *Gallus* species. In particular, we observed a surprisingly high weighting (~ 18–19%) for topology 9 (T9), almost as high as the tree species topology (T12, ~ 20%) (Fig. 3). Moreover, Treemix result (Additional file 2: Figure S1B) also supports ancestral admixture between the Ceylon/Grey junglefowl lineage and the Green junglefowl one. All these results are indicative of incomplete lineage sorting and/or introgression during the history of the genus. While the three non-red junglefowls (i.e. Grey, Ceylon, and Green) are allopatric, the fluctuating climatic changes of the Pliocene and early Pleistocene geological era may have not only triggered speciation events within the genus but could have also led to subsequent geographic contact between incipient species providing opportunities for hybridisation.

∂a∂i estimation of divergence time between domestic chicken and Red Junglefowl is 10 times older than our direct estimate based on absolute pairwise sequence divergence. The domestication history of the chicken remains debatable with the contribution of one [3] or several subspecies of Red junglefowl [4]. It is possible that the main ancestral Red junglefowl subspecies population were not represented. The Red junglefowls in this study are all from Southeast Asia and do not include any representative from the Indian subcontinent. For the direct estimation approach, we considered the aggregate of nucleotide diversity among the subspecies that have accumulated over different evolutionary timescales in estimating the domestication period of the chicken. However, ∂a∂i is assuming that the Red junglefowl samples represent a homogenous population. Accordingly, the ~ 81 KYA estimation by ∂a∂i might include both the time since chicken domestication and the earlier split times among the Red junglefowl subspecies. Considering the commensal mode of domestication proposed for the species [29], the time of chicken domestication would be unlikely older than the time spanning the beginning of farming and human settlements, which started in the Neolithic (10,000–12,000 years ago). Therefore, ~ 8000 years ago is the most realistic estimation. Calculation of the divergence time estimation between domestic chicken and each of the four subspecies of Red junglefowl, particularly *G. g. murghi* from the Indian subcontinent, not included in this study, may further clarify the issue of the domestication time of the species.

The phylogenies of the genus *Gallus* reported here differ from those in other studies [30–32], which are based on short fragments of the genome. In particular, we show here a sister relationship between the Grey and the Ceylon junglefowls, rather than between the Grey and the Red junglefowls [30, 32] or between the Green and the Red junglefowls [31]. A sister relationship between the Grey and the Ceylon junglefowls agrees with the current geographic distribution of these two species in South India and Ceylon (Sri Lanka), respectively. Other studies also indicate more ancient divergence times between the different *Gallus* lineages than the ones reported here (see TimeTree [33]). For example, the separation between the Grey and the Ceylon junglefowls ~ 1.8 MYA (CI 1.52–1.91 MYA) in this study is more recent than the 8.05 MYA (CI 3.94–12.15 MYA) reported by TimeTree [33]. Several reasons for such discrepancy may be advocated, e.g. the use of full genome information rather than the fragmentary ones as well as different mean Galliforme neutral mutation rates between studies.

Several lines of evidence support recent introgression into domestic chicken from other *Gallus* species: (i) Within candidate introgressed fragments, we observe an excess of sequences sharing variation between the donors and recipient species, low absolute divergence index with the donor species, and genealogical nesting of the candidate introgressed haplotypes within or close to the donor species in both the phylogenies and networks analyses; (ii) comparison of the *D*-statistic for the autosomes and the Z chromosome show higher levels of admixture on the former than the latter. This trend is not unusual for introgression between species, as species barriers to introgression are often stronger on the sex chromosomes compared to the autosomes [34]; (iii) we report large genomic tracts of introgression, larger than expected if it results from incomplete lineage sorting. It is consistent with recent introgression events where the introgressed haplotypes have not yet been broken down by recombination [35, 36]. Together, all these evidences strongly support that the candidate introgression reported here represent true introgressed regions from the three non-red junglefowl species into the domestic chicken.

Our results also show extensive introgression from domestic chicken/Red junglefowl into Grey junglefowl with introgressed tracts up to 26 Mb in size. It supports recent introgression events in the Grey junglefowl examined here, which originate from a captive bred population. The close relationship between the domestic chicken and the Red junglefowl makes it difficult to pinpoint the source (domestic or Red junglefowl) of these introgressed alleles in the Grey junglefowl. Specifically, the introgression in the Grey junglefowl might have originated in the wild from the Red junglefowl or it might have followed the domestication and the dispersion of domestic chicken, considering the long history of sympatry between the domestic chicken and the Grey junglefowl across India. Detailed genome analysis of candidate introgressed regions in the wild Grey junglefowl as well as the inclusion, in further studies, of the Red junglefowl subspecies from the Indian subcontinent *G. g. murghi* may further clarify these issues. Interestingly, among the introgressed haplotype regions in the Grey junglefowl, we found several previously proposed chicken domestication genes (e.g. *DACH1*, *RAB28*) [37, 38] supporting domestic chicken introgression events. Our results highlight the need for further studies of wild Grey junglefowl populations to assess whether their genetic integrity is being threatened by domestic chicken introgression.

We identified introgression from the Grey junglefowl into all but the Langshan domestic chicken populations. Considering the geographic distribution of the Grey junglefowl, it supports that the domestic chickens were initially introgressed with this species on the Indian subcontinent prior to their dispersion towards Africa (Ethiopia), the Arabian Peninsula (Saudi Arabia), Sri Lanka, Indonesia, and Europe. Interestingly, Ethiopia is the region with the largest proportion of introgressed Grey junglefowl haplotypes in domestic chicken (Additional file 8: Table S3), possibly a legacy of direct trading routes between the Southern part of the Indian subcontinent and East Africa. Surprisingly, we also find evidence of Grey junglefowl introgression into one of the wild Red junglefowl. This Red junglefowl sample originated from the Yunnan Province in China [39], well outside the geographic distribution of the Grey junglefowl confined to India. Such signature of introgression is likely the result of crossbreeding between domestic chicken and local wild Red junglefowl. Introgression between domestic chicken and wild Red junglefowl has been shown in the past using microsatellite loci in Vietnam [40]. By extension, this result supports a movement of domestic chicken from the centre of origin on the Indian subcontinent towards East and Southeast Asia. This hypothesis is also supported by mtDNA analysis which indicates the presence, at low frequency, of a mtDNA haplogroup in East Asia likely originated from the Indian subcontinent [4].

Our results also highlight the limitations of the current approaches for introgression analysis when dealing with closely related species. Hence, the need to include all candidate donor species for the correct interpretation of the introgression patterns, and the importance to complement the genome-wide analysis of introgression with locus-specific studies including phylogenetic analysis of haplotypes. The *Gallus* species phylogeny indicates that the Grey and the Ceylon junglefowls are sister species, which speciated before the separation of the Red junglefowl/domestic chicken lineages. The detailed analysis of candidate introgressed regions reveals that the majority of the Ceylon junglefowl candidate *f*_*d*_ correspond to introgression events involving the Grey junglefowl. It highlights the limitation of both the genome-wide *D*-statistics and local admixture proportion estimates when there are multiple closely related donor species. Only a detailed assessment of all the significant *f*_*d*_ candidates using multiple statistics allowed us to identify regions showing introgression from Ceylon junglefowl into the domestic chicken.

At the scale of individual candidate regions, we also observe a different pattern of introgression for the Grey and the Ceylon junglefowls. While we identify several strong cases of introgression from the Grey junglefowl into the domestic chicken, evidence for Ceylon junglefowl introgression are limited to one or two Sri Lankan domestic haplotypes at each introgressed region. Similarly, we only reveal one case of introgression from the domestic chicken into wild Ceylon junglefowl, a somewhat surprising result considering the sister relationship between the Ceylon and the Grey junglefowls. While we cannot exclude a sampling artefact, the findings suggest that the impact of introgression from Ceylon junglefowl into the domestic chicken might be restricted to the Sri Lankan domestic chicken. Fertile hybrids between the Ceylon junglefowl with both the Red and the Grey junglefowls have been bred in captivity [5]. There is also anecdotal evidence of human-mediated crosses between male Ceylon junglefowl and female domestic chicken in Sri Lanka to increase the cockfighting vigour of roosters (Pradeepa Silva personal communication) [9].

Crosses between the Green junglefowl and domestic chicken are common in Indonesia [5], and the estimations of admixture proportion (*f*) between the domestic chicken and the Green junglefowl are ~ 9% and ~ 7% for the autosomes and the Z chromosome, respectively (Table 3). However, our results support only a single compelling example of introgression from the Green junglefowl into the domestic chicken. This signal is limited to the Langshan, a Chinese chicken breed. It may represent a legacy of the movement of domestic birds from the Indonesian Islands to the East Asian continent. However, no candidate introgressed regions were detected in the Indonesian domestic chickens (Kedu Hitam and Sumatra). Analyses of more Indonesian domestic chicken populations are therefore required.

There are increasing evidence for “adaptive” cross-species introgression among mammalian domesticates [41] as well as in humans [36]. A previous study has reported that the chicken yellow skin phenotype is the consequence of introgression event(s) from the Grey junglefowl into the domestic chicken [10], a phenotype favoured by some chicken breeders and now fixed in several fancy and commercial breeds [10, 38]. Here, besides some traditional monomorphic breeds (e.g. Langshan, Kedu Hitam, and Sumatra), we analysed village chicken populations that are typically characterised by a high level of phenotypic diversity (e.g. plumage colour and pattern, morphology). Introgressed regions were not found fixed or approaching fixation in any of the indigenous village chicken populations examined. Undoubtedly, these candidate introgressed regions contribute to the genome diversity of the domestic chicken, and while we have no evidence of positive selection at these introgressed regions [37], other selection pressures (e.g. heterozygote advantage—balancing selection) may be acting. How many of these introgressions have influenced the phenotypic diversity of these village chickens remains unclear.

Examples of genes within introgressed regions from the Grey junglefowl in the domestic chicken are *NOX3* and *GSC*, which are involved in the ear development and biogenesis of otoconia supporting balance and gravity detection [42, 43]; *CPEB3*, which is associated with thermoception and enhancing memory [44, 45] and could play a central role in adaptation to new environments; *MME*, which plays a role in stimulating cytokine production [46]; and *RAP2B*, which is mainly expressed in the neutrophils for platelet activation and aggregation [47]. Other genes of interests include *CDC5L* and *FOXP2* introgressed from the Ceylon junglefowl. The former is a key mitotic progression regulator involved in DNA damage response [48], and the latter is a gene involved in song learning in birds [49]. *IPO7*, which is introgressed from the Green junglefowl, plays a role in the innate immune system [50].

## Conclusions

Our study reveals a polyphyletic origin of domestic chicken diversity with the Red junglefowl as the main ancestor and subsequent introgression from the Grey, Ceylon, and Green junglefowls. These findings provide new insights into the domestication and evolutionary history of the species. Considering the present geographic distributions of the non-red junglefowl species and the dispersal history of the domestic chickens, the level of introgression among domestic populations will be expected to vary from one geographic region to another. Analysis of domestic chicken populations on a wider geographic scale may provide us with a detailed map of the presence and frequency of introgressed genome regions. Our results shed new lights on the origin of the diversity of our most important agricultural livestock species, and they illustrate the uniqueness and diversity of each local domestic chicken population across the world.

## Materials and methods

### Sampling and DNA extraction

Sample information (*n* = 87) including their geographic location is provided in Additional file 1: Table S1. Blood samples were collected from the wing vein of 27 indigenous village domestic chickens from 3 countries (i.e. Ethiopia (*n* = 11), Saudi Arabia (*n* = 5), and Sri Lanka (*n* = 11)) [9, 37, 51], 8 Chinese Langshan chicken sampled in the UK, and 11 non-red junglefowl *Gallus* species (i.e. Grey (*n* = 2), Ceylon (*n* = 7), and Green (*n* = 2) junglefowls). Blood samples from five of the Ceylon junglefowl were obtained from the wild in Uva province of Sri Lanka, while the remaining two Ceylon junglefowl blood were sampled from Koen Vanmechelen's collection. The two common Pheasants, *Phasianus colchicus*, were sampled from the wild in the UK. Genomic DNA was extracted following the standard phenol-chloroform extraction procedure method [52]. Genome sequencing was performed on the Illumina HiSeq 2000/2500/X platforms with an average depth of 30× coverage.

This dataset was complemented with genome sequences from two domestic fancy chicken breeds (Poule de Bresse and Mechelse Koekoek), one Mechelse Styrian, a 16th generation crossbred bird from the Cosmopolitan Chicken Research Project (CCRP) [53], and one Red, Grey, Ceylon, and Green junglefowl sequences also from Koen Vanmechelen collection [53]. The publicly retrieved genome sequences of 15 Indonesian indigenous chickens (Sumatra, *n* = 5, and Kedu Hitam, *n* = 10) [54], 3 Javanese red junglefowls *G. g. bankiva* and 9 Green junglefowls [54], and 5 Red junglefowls, sampled in Yunnan or Hainan Provinces (People’s Republic of China) [39], were included in our study. The genome sequence depth for these birds ranges from 8× to 14×.

In total, these 87 genomes include 53 domestic chickens, 6 Red junglefowls, 3 Javanese red junglefowls, 3 Grey junglefowls, 8 Ceylon junglefowls, 12 Green junglefowls, and 2 common Pheasants.

### Sequence mapping and variant calling

Raw reads were trimmed of adapter contamination at the sequencing centre (i.e. BGI/Edinburgh Genomics), and reads that contained more than 50% low quality bases (quality value ≤ 5) were removed. Reads from all genomes were mapped independently to the *Galgal* 5.0 reference genome [55] using the Burrows-Wheeler Aligner bwa mem option version 0.7.15 [56], and duplicates were marked using Picard tools version 2.9.0 [57]. Following the genome analysis toolkit (GATK) version 3.8.0 best practises [58], we performed local realignment around INDELs to minimise the number of mismatching bases across all reads. To apply a base quality score recalibration step to reduce the significance of any sequencing errors, we used a bootstrapping approach across both the wild non-red junglefowl species and common Pheasant that has no known sets of high-quality database SNPs. We applied the same approach to the Red junglefowl for consistency. To do this, we ran an initial variant calling on individual unrecalibrated BAM files and then extracted the variants with the highest confidence based on the following criteria: --filterexpression “QD < 2.0 || FS > 60.0 || MQ < 40.0.” We then used this high-quality set of SNPs as the input for the known set of database SNPs. Finally, we performed a variant on the recalibrated data. We repeated these steps in a loop for multiple times until convergence was reached for each sample.

To improve the genotype likelihoods for all samples using standard hard filtering parameters, we followed the multisample aggregation approach, which jointly genotypes variants by merging records of all samples using the “-ERC GVCF” mode in “HaplotypeCaller.” We first called variants per sample to generate an intermediate genomic (gVCF) file. Joint genotype was performed for each species separately using “GenotypeGVCFs” and then subsequently merged with BCFtools version 1.4 [59]. Variants were called using Hard filtering --filterExpression “QD < 2.0 || FS > 60.0 || MQ < 40.0 || MQRankSum < -12.5 || ReadPosRankSum < -8.0.” All downstream analyses were restricted to the autosomes, the Z chromosome, and the mitochondrial DNA. The percentage of the mapped reads and read pairs properly mapped to the same chromosome were calculated using SAMtools “flagstat” version 1.4 [59] while the number of SNPs per sample was identified using VCFtools “vcf-stats” version 0.1.14 [60].

### Population genetic structure

Principal component analysis was performed on the SNPs identified across the autosomes, filtered with “--indep-pairwise 50 10 0.3,” to visualise the genetic structure of the junglefowl species using PLINK version 1.9 [61]. Admixture analysis using ADMIXTURE version 1.3.0 [62] was performed unsupervised for 5 fold cross-validation for 1 through 5 clusters (*K*).

### Species tree

To unravel the species tree of the genus, we constructed an autosomal neighbour-joining phylogenetic tree using Phyml version 3.0 [63] and network using NeighborNet option of SplitsTree version 4.14.6. First, the dataset was filtered to sites separated by at least 1 kb and then converted to a PHYLIP sequence file using publicly available scripts [64]. We also constructed a maximum likelihood tree on the exon variants. This was done by first annotating the entire whole-genome VCF file with SnpEff and then extracting different variants effect within the exons using SnpSift [65]. As with the above, all trees including the Z chromosome were based on polymorphic sites but not for the mtDNA (i.e. all consensus sequences were used). All trees were plotted using the General Time Reversible (GTR) model of nucleotide substitution following its prediction by jModeltest 2.1.7 [66] and then viewed in MEGA 7.0 [67].

After phasing all the autosomal SNPs using SHAPEIT [68], we next performed “Topology Weighting by Iterative Sampling of Sub-Trees” (*Twisst*) [22], which summarised the relationships among multiple samples in a tree by providing a weighting for each possible sub-tree topology. Neighbour-joining trees were generated for windows containing exactly 50 SNPs using Phyml 3.0 [63]. Topologies were plotted in R using the package “APE” version 5.1 [69]. We ran the TreeMix [70] with a block size of 1000 SNPs per window after having filtered the VCF file with “maf 0.01” using PLINK version 1.9 [61].

### Species divergence time

We used two approaches for the estimation of divergence time between species. We first measured the autosomal average absolute pairwise sequence divergence between each species pair using the equation below. This measure represents the sum of accumulated divergence since speciation and pairwise differences existed in the ancestral population [71].

$$ T=K/2r $$

where *K* is the average sequence divergence for pairwise species. We included both the variant and non-variant sites from the autosomes in the analysis of *K*, which was run in every 100 kb region of the genome with 20 kb step size. *r* is the Galliformes nucleotide substitution rate per site per year 1.3 (1.2 − 1.5) × 10^−9^ [72], and *T* is the time in years.

To estimate the species split time, we adjusted this measure of divergence downward by subtracting an estimated ancestral diversity, which we took as the average diversity (π) of the two daughters’ species (i.e. *d*_*a*_ [23]) using the equation below. The estimated divergence time is reported in years, assuming one generation per year.

$$ T=\left(K-\uppi \right)/2r $$

Using the most common species topology, the average π = (π_Pheasant_ + (π_Green_ + ((π_Grey_ + π_Ceylon_)/2 + (π_Javanese Red_ + π_Red_)/2)/2)/2.

For the model-based inference using ∂a∂i [73], we generated the input (folded) 2D site frequency spectrum (SFS) using ANGSD [64] directly from the BAM file, producing an SFS representing at least 1.01 billion sites. We then fitted a model that included parameters for the population size of each species, the split time, and the migration rates in each direction. We repeated the optimisation procedure 50 times to ensure that maximum likelihood parameters were found, and we also confirmed that using different starting values and upper and lower bounds for the optimisation process did not alter the final parameter estimates. For the Ceylon and the Green junglefowls, we ran an additional model that included heterogenous effective population size (with two classes of loci shared by the two populations to account for selection at linked sites affecting local *N*_e_) [74].

### Estimating tract lengths for shared haplotypes under incomplete lineage sorting

Using the approach of Huerta-Sánchez et al. [75], we estimated the likely length of shared haplotypes across the genome following incomplete ancestral lineage sorting. This was done with the equation:

$$ L=1/\left(r\times t\right) $$

where *L* is the expected length of a shared ancestral sequence, *r* is the recombination rate per generation per bp (3 × 10^−8^ for chicken on the autosomes) [76], and *t* is the expected divergence time across the junglefowl (~ 4 MYA), assuming 1 year generation time.

### Detecting introgression

First, we computed *D*-statistics [24, 25] to test for a genome-wide excess of shared derived allele(s) between two in-groups using the outgroup as representative of the ancestral state. Considering the three in-groups, *P*_*1*_ (Red junglefowl), *P*_*2*_ (domestic chicken), and *P*_*3*_ (Grey or Ceylon or Green junglefowl), and an out-group *O* (common Pheasant), the expected phylogeny is (((*P*_*1*_, *P*_*2*_), *P*_*3*_), *O*). ABBA denotes sites where the derived allele “B” is shared between the domestic chicken “*P*_*2*_” and the Grey or Ceylon or Green junglefowl “*P*_*3*_,” while the Red junglefowl “*P*_*1*_” shares the ancestral allele “A” with the common Pheasant “*O*.” BABA denotes sites where the Red junglefowl “*P*_*1*_” shares the derived allele ^“^B^”^ with “*P*_*3*_” while the domestic chicken “*P*_*2*_” shares the same ancestral state with the outgroup “*O*.” The majority of ABBA and BABA patterns are due to incomplete lineage sorting, but an excess of one over the other can be indicative of introgression [24–26]. *D* is the relative excess computed as the difference in the number of ABBA and BABA sites divided by the total number of ABBA and BABA sites. Under the assumption of no gene flow and a neutral coalescent model, counts of both ABBA and BABA should be similar and *D* should tend towards zero. We used the approach of Durand et .al [25] to compute ABBA and BABA counts from allele frequencies, in which each SNP contributes to the counts even if it is not fixed. We used the jackknife approach with a block size of 1 Mb to test for a significant deviation of *D* from zero (i.e consistent with introgression), using a minimum *Z*-score of 4 as significant. We then estimated the proportion of admixture, *f* [24, 25].

### Identifying introgression at particular loci and inferring the direction of introgression

To identify specific regions showing introgression between the domestic chicken and the non-red junglefowl species, we used a combination of analyses. First, we estimated *f*_*d*_ [26], which is based on the four-taxon ABBA-BABA statistics and which was designed to detect and quantify bidirectional introgression at particular loci [26]. *f*_*d*_ was computed in 100 kb windows with a 20-kb step size. Each window was required to contain a minimum of 100 SNPs. No threshold value was used to avoid excluding peaks which may have introgressed only a few domestic chickens. Rather, we decided to analyse each of them exhaustively (see Raman Akinyanju Lawal PhD thesis [9] for further details). These *f*_*d*_ regions were then extracted and further investigated using *Twisst* [22] to test for a deviation in topology weightings in the candidate regions. Here, we used only four taxa: domestic chicken, Red junglefowl, common Pheasant, and either the Grey, Ceylon, or Green junglefowl.

Next, we constructed haplotype-based gene trees and networks to make inferences about the direction of gene flow. The expectation is that introgressed regions in domestic chicken from any of the non-red junglefowl will be indicated by finding chicken haplotypes nested within the donor species, or with the donor species haplotypes at the root of the introgressed ones. For regions in non-red junglefowl that are introgressed from domestic chicken, the expectation is that the introgressed haplotypes will be nested within the domestic chicken clade. Sequences from the candidate introgressed regions were phased using SHAPEIT [68]. The phased haplotypes were converted into a VCF file and subsequently formatted in Plink 1.9 [77] with the “beagle recode” option, the output from which was provided as an input to a custom bash script to generate a FASTA file. The optimal molecular evolutionary model was inferred using jModeltest 2.1.7 [66] based on the Akaike information criterion (AIC). Phyml 3.0 [63] was used to compute the approximate likelihood ratio score for each branch using the best predicted model. For the network, we used the NeighborNet option of SplitsTree version 4.14.6. The input file for the network was a distance matrix created using “distMat.py” accessible at [64].

Finally, we examined levels of divergence between species to further validate our candidate regions. Introgression between domestic chicken and either the Grey, Ceylon, or Green junglefowl is expected to reduce genetic divergence between the two species, regardless of the direction of introgression. Introgression into domestic chicken is expected to also increase divergence between domestic chicken and Red junglefowl, whereas introgression from domestic chicken into the Grey, Ceylon, or Green junglefowls should not affect divergence between domestic chicken and Red junglefowl. We therefore computed relative (*F*_*ST*_) and absolute (*d*_*XY*_) measures of divergence between pairs using the script “popgenWindows.py” [64].

### Remapping of candidate introgressed regions to GRCg6a

Following the recent release of a new reference genome (GRCg6a), all candidate introgressed regions obtained from *Galgal* 5.0 were remapped using the NCBI remapper tool. All remapping options were set to the default threshold. Only the GRCg6a coordinates for the candidate introgressed regions and genes are reported here throughout the manuscript.

## Supplementary information

**Additional file 1: Table S1**: Sampling, mapping and variants statistics. HomAA and HetRA are the proportion of homozygous and heterozygous SNPs to the reference (*Galgal*5.0), respectively.

**Additional file 2: Figure S1. A** Maximum likelihood tree generated from 1,849,580 exon SNPs with GTR model. All branches are supported by 100% bootstrap values. **B** TreeMix across the autosomal genome. **C.** *Twisst* for Grey, Ceylon, Green, Red junglefowls and domestic chicken. The numbers above each bar are the proportion of admixture for that topology expressed in percentage.

**Additional file 3: Figure S2.** The yellow skin locus (Chr24: 6107101–6,135,115 bp) for the introgression from the Grey junglefowl to some domestic chicken. **A** *f*_*d*_ plot, **B** *Twisst* plot, **B**_**1**_ its topologies and **B**_**2**_ their proportions. **C** *dxy* and **D** *Fst* . Eth, Sau, SriLanka, SE + E are domestic chickens from Ethiopia, Saudi Arabia, Sri Lanka and Southeast Asia (Indonesia) and East Asia (China), respectively. **E** maximum likelihood tree.

**Additional file 4 : Table S2**. Candidate introgressed regions from domestic chicken/Red junglefowl into Grey/Ceylon junglefowl. *Positions along the chromosome in megabase (Mb).

**Additional file 5: Figure S3.** A 26 Mb introgressed region on chromosome 1 (141287737–167,334,186 bp).
The following description is applicable to the Additional files 5, 6, 7 which show figures for the introgressed regions from the domestic chicken into Grey junglefowl. **A** *fd* plot for the introgressed chromosome, **B** maximum likelihood tree for the introgressed region and **C** haplotype-based network, **D** *Twisst* plot and the proportion for each of the three possible topologies in the introgressed regions, **E** *d*_*XY*_ and **F** *F*_*ST*_. Eth, Sau, SriLanka, Lang, Ked, Sum represent chicken samples from Ethiopia, Saudi Arabia, Sri Lanka, Langshan (China), Kedu Hitam and Sumatra (Indonesia), respectively, GreyJ represents Grey junglefowl, and targetGreyJ are the introgressed (*) Grey junglefowl haplotypes. Domestic includes all the domestic chicken populations. Common Pheasant, the outgroup, was intentionally excluded from **Figure S3** and **S4** trees due to the large length of the region.

**Additional file 6: Figure S4.** A 9 Mb introgressed region on chromosome 2 (11022874–19,972,089 bp). See description for this file under Additional file 5 above.

**Additional file 7: Figure S5**. A 2.8 Mb introgressed region on chromosome 4 (76429662–79,206,239 bp). See description for this file under Additional file 5 above.

**Additional file 8: Table S3**. Candidate introgressed regions from non-red junglefowl into domestic chicken/Red junglefowl. *Positions along the chromosome in megabase (Mb), **SEA (Southeast and East Asia) (see methods for sampling location), ***Ensembl release version 96.

**Additional file 9: Figure S6.** A 220 kb (Chr 2: 119676880–119,901,132 bp) introgressed region from Grey junglefowl into domestic chicken. targetDom here include the introgressed domestic chicken and a single Red junglefowl haplotypes (*).
The following description is applicable to the Additional files 9, 10, 11, 12, 13, 14 and 15 which show figures for the introgressed regions from the Grey junglefowl to domestic chicken/Red junglefowl. The plots are zoomed close to the region. **A** *f*_*d*_ plot, **B** haplotype-based network and **C** maximum likelihood tree for the introgressed region. **D** *Twisst* plot and **D**_**1**_ its proportion for each of the three possible topologies in the introgressed region. **E** *d*_*XY*_ and **F** *F*_*ST*_. Eth, Sau, SriLanka, Lang, Ked, Sum represent chicken samples from Ethiopia, Saudi Arabia, Sri Lanka, Langshan (China), Kedu Hitam and Sumatra (Indonesia), respectively. GreyJ represent Grey junglefowl, and targetDom are the introgressed (*) domestic haplotypes.

**Additional file 10: Figure S7.** A 100 kb (Chr 3: 50759656–50,859,645 bp) introgressed region from Grey junglefowl into domestic chicken. See description for this file under Addtional file 9 above.

**Additional file 11: Figure S8.** A 200 kb (Chr 4: 62097304–62,297,319 bp) introgressed region from Grey junglefowl into domestic chicken. See description for this file under Addtional file 9 above.

**Additional file 12: Figure S9.** A 280 kb (Chr 5: 45674368–45,954,418 bp) introgressed region from Grey junglefowl into domestic chicken. See description for this file under Addtional file 9 above.

**Additional file 13: Figure S10.** A 140 kb (Chr 7: 22652767–22,792,759 bp) introgressed region from Grey junglefowl into domestic chicken. See description for this file under Addtional file 9 above.

**Additional file 14: Figure S11.** A 500 kb (Chr 9: 23052049–23,552,045 bp) introgressed region from Grey junglefowl into domestic chicken. See description for this file under Addtional file 9 above.

**Additional file 15: Figure S12.** A 100 kb (Chr 12: 12914268–13,014,266 bp) introgressed region from (*) Grey junglefowl into domestic chicken and (**) from domestic chicken to Grey junglefowl. The *Twisst* values and plots are based on the introgressed domestic haplotypes from the Grey junglefowl and do not account for the reverse introgression. See description for this file under Addtional file 9 above.

**Additional file 16: Figure S13.** The *fd* plots test for the comparison between Ceylon junglefowl and domestic chicken population from (**A**) Ethiopia and Saudi, (**B**) Sri Lanka and (**C**) Southeast and East Asia. The *Y*-axis fd value and X-axis 1–28 autosomes.

**Additional file 17: Figure S14.** Network and *Twisst* proportion of topologies for three Ceylon candidate introgressed regions into domestic chicken (**A - C**). (**A1)** and (**A2**) 6.52 Mb region Chr 1: 2895616–9,418,660 bp, (**B1**) and (**B2**) 3.95 Mb Chr 1: 25261354–29,205,161 bp, (**C1**) and (**C2**) 1.38 Mb region Chr 1: 147936229–149,316,591 bp. **C1** also shows support for (*) introgression from domestic chicken to some Grey junglefowl haplotypes at the same region.

**Additional file 18: Figure S15.** A 600 kb (Chr 3: 108325801–108,925,723 bp) introgressed region from Ceylon junglefowl to domestic chicken. **A** *f*_*d*_ plot, **B** haplotype-based network, **C** maximum likelihood tree, **D** *Twisst* plot and **D**_**1**_ its proportion for each of the three possible topologies, **E** *d*_*XY*_ and **F** *F*_*ST*_. Eth, Sau, SriLanka, Lang, Ked, Sum represent chicken samples from Ethiopia, Saudi Arabia, Sri Lanka, Langshan (China), Kedu Hitam and Sumatra (Indonesia), respectively. CeylonJ is Ceylon junglefowl and targetDom are the introgressed domestic chicken haplotypes (*).

**Additional file 19: Figure S16.** The *fd* plots test for the comparison between Green junglefowl and domestic chicken population from (**A**) Ethiopia and Saudi, (**B**) Sri Lanka and (**C**) Southeast and East Asia. The Y-axis fd value and X-axis 1–28 autosomes.

**Additional file 20: Figure S17:** A 100 kb (Chr 5: 9538715–9,638,713 bp) introgressed region from Green junglefowl into domestic chicken. **A** *f*_*d*_ plot, **B** haplotype-based network, **C** maximum likelihood tree, **D** *Twisst* plot and **D**_**1**_ its proportion for each of the three possible topologies. **E** *d*_*XY*_ and **F** *F*_*ST*_. Eth, Sau, SriLanka, Lang, Ked, Sum represent chicken samples from Ethiopia, Saudi Arabia, Sri Lanka, Langshan (China), Kedu Hitam and Sumatra (Indonesia), respectively. GreenJ is Green junglefowl and targetDom are the introgressed domestic haplotypes (*).

## Data Availability

Sequence data generated by this study is available at https://www.ncbi.nlm.nih.gov/sra/PRJNA432200 or in the NCBI with the accession number PRJNA432200 [78]. We also included sequences from our most recent study found at https://www.ncbi.nlm.nih.gov/sra/SRP142580 or in the NCBI with the accession number SRP142580 [37, 79]. Additional sequence data available from other studies [39, 54, 80].
