# Supplementary figures and images for: The wild species genome ancestry of domestic chickens
Source: BMC Biol. 2020 Feb 12;18:13. doi: 10.1186/s12915-020-0738-1 (PMC7014787; doi:10.1186/s12915-020-0738-1)

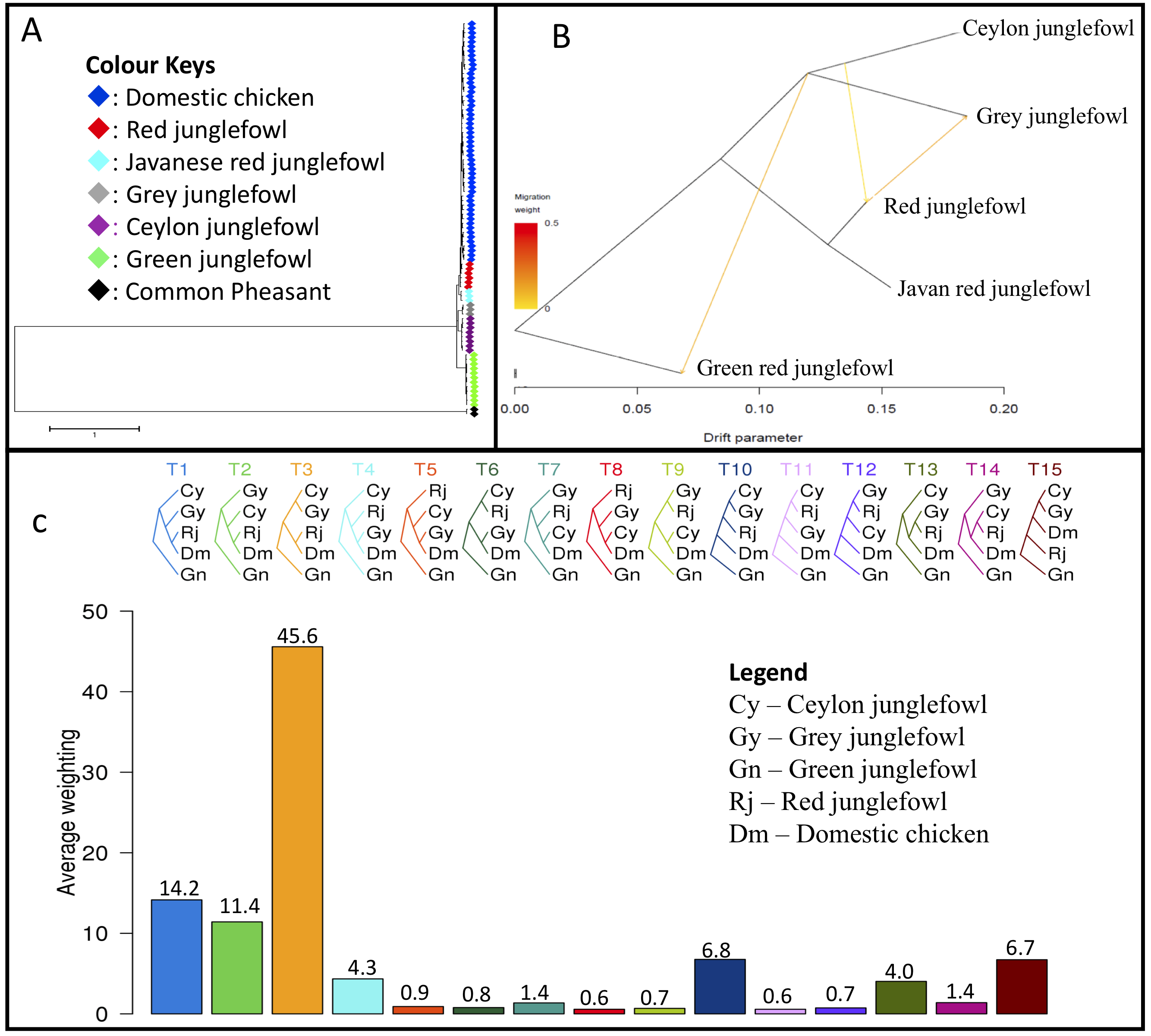

Supplement: Supplementary file 2 — Additional file 2: Figure S1. A Maximum likelihood tree generated from 1,849,580 exon SNPs with GTR model. All branches are supported by 100% bootstrap values. B TreeMix across the autosomal genome. C. Twisst for Grey, Ceylon, Green, Red junglefowls and domestic chicken. The numbers above each bar are the proportion of admixture for that topology expressed in percentage. [file 12915_2020_738_MOESM2_ESM.tiff]

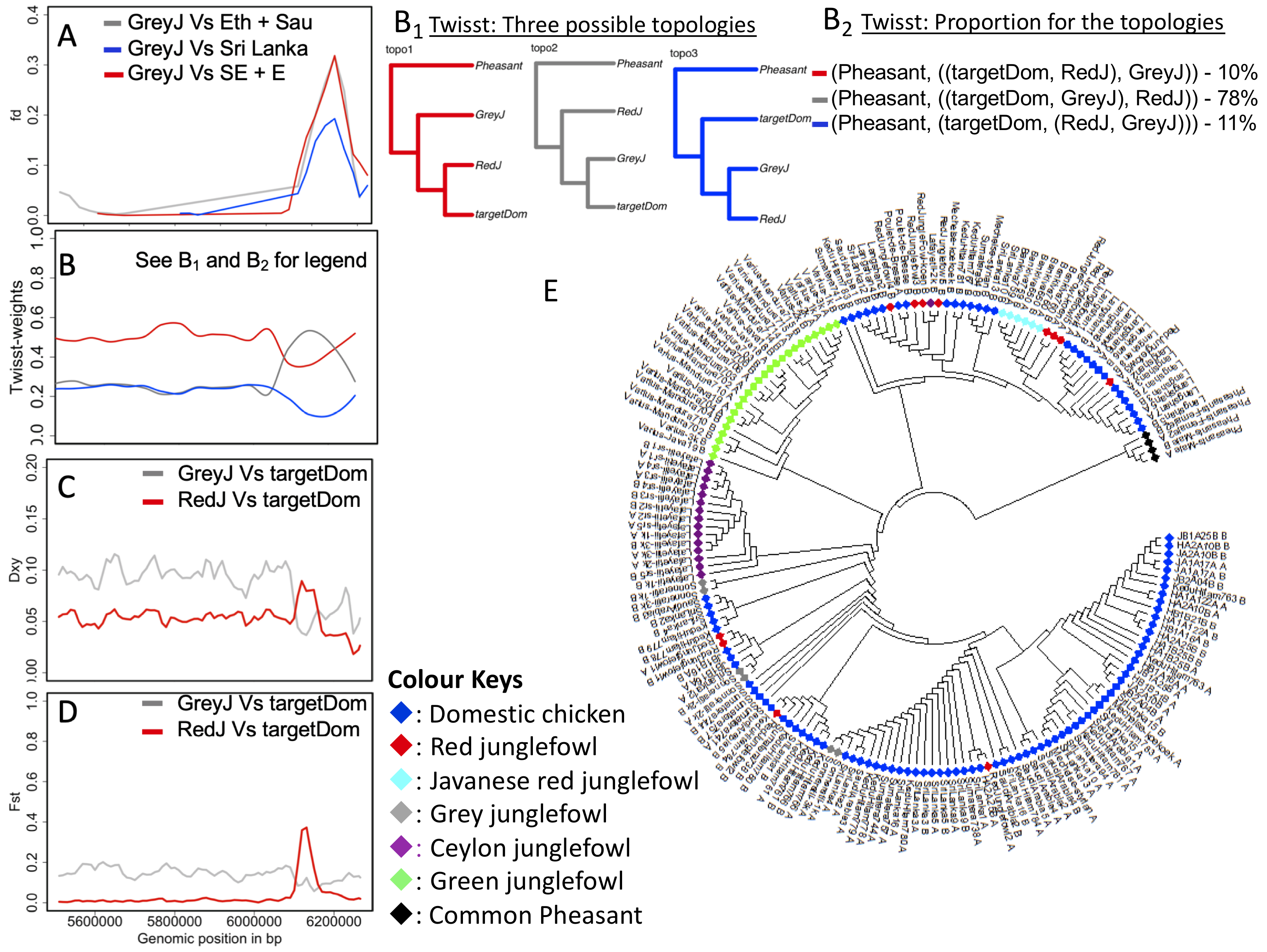

Supplement: Supplementary file 3 — Additional file 3: Figure S2. The yellow skin locus (Chr24: 6107101–6,135,115 bp) for the introgression from the Grey junglefowl to some domestic chicken. A fd plot, B Twisst plot, B1 its topologies and B2 their proportions. C dxy and D Fst . Eth, Sau, SriLanka, SE + E are domestic chickens from Ethiopia, Saudi Arabia, Sri Lanka and Southeast Asia (Indonesia) and East Asia (China), respectively. E maximum likelihood tree. [file 12915_2020_738_MOESM3_ESM.tiff]

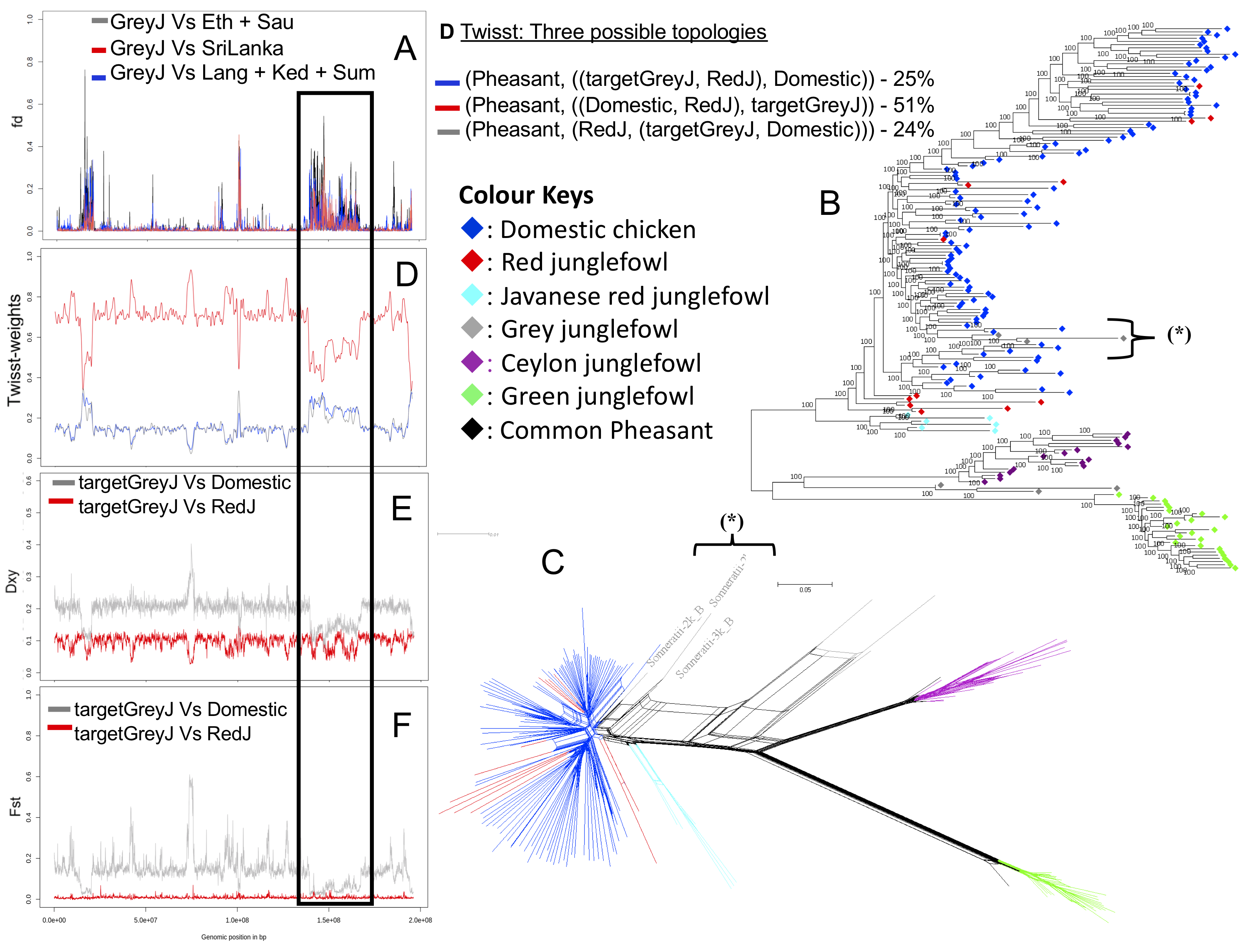

Supplement: Supplementary file 5 — Additional file 5: Figure S3. A 26 Mb introgressed region on chromosome 1 (141287737–167,334,186 bp). The following description is applicable to the Additional files 5, 6, 7 which show figures for the introgressed regions from the domestic chicken into Grey junglefowl. A fd plot for the introgressed chromosome, B maximum likelihood tree for the introgressed region and C haplotype-based network, D Twisst plot and the proportion for each of the three possible topologies in the introgressed regions, E dXY and F FST. Eth, Sau, SriLanka, Lang, Ked, Sum represent chicken samples from Ethiopia, Saudi Arabia, Sri Lanka, Langshan (China), Kedu Hitam and Sumatra (Indonesia), respectively, GreyJ represents Grey junglefowl, and targetGreyJ are the introgressed (*) Grey junglefowl haplotypes. Domestic includes all the domestic chicken populations. Common Pheasant, the outgroup, was intentionally excluded from Figure S3 and S4 trees due to the large length of the region. [file 12915_2020_738_MOESM5_ESM.tiff]

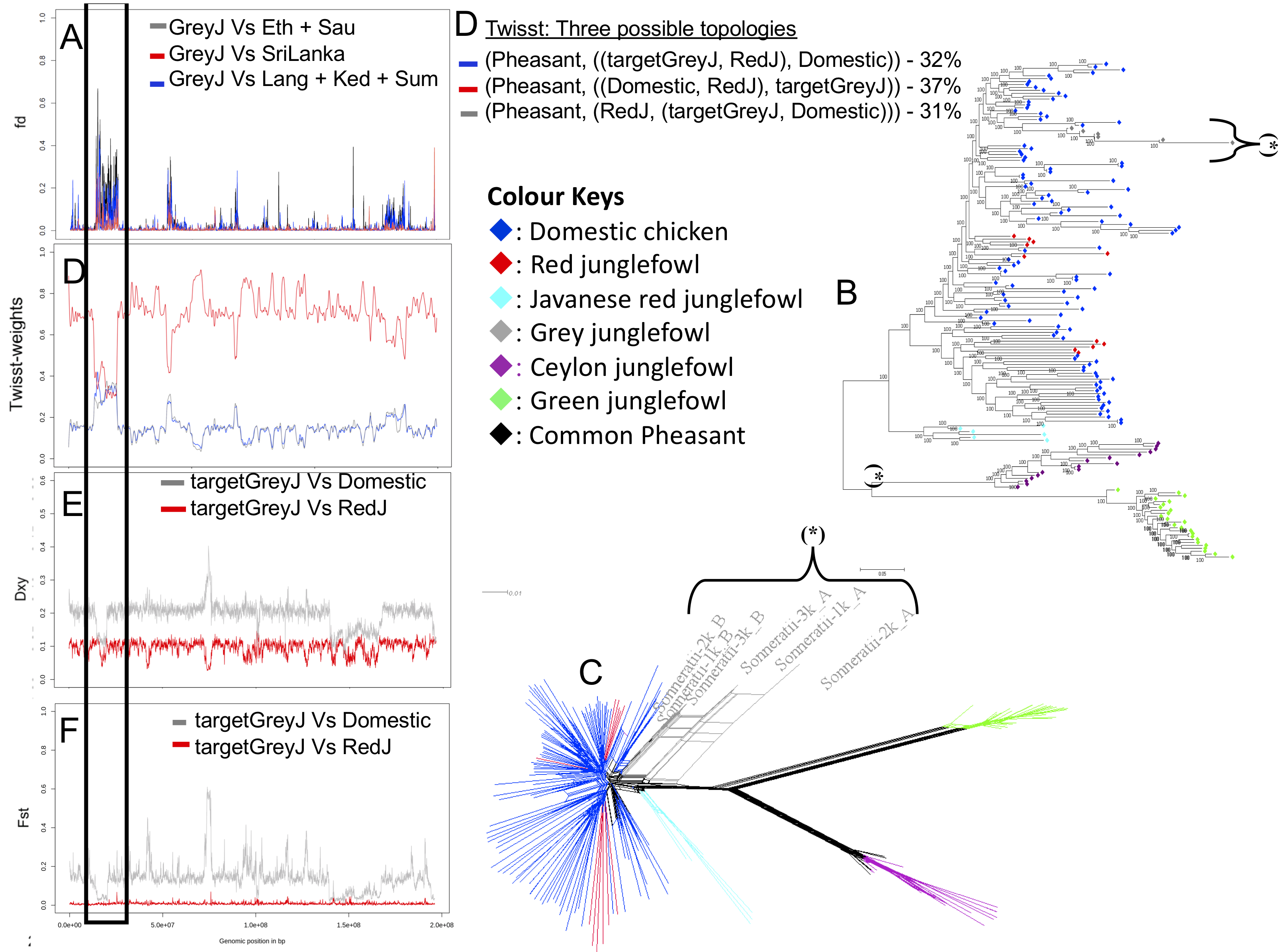

Supplement: Supplementary file 6 — Additional file 6: Figure S4. A 9 Mb introgressed region on chromosome 2 (11022874–19,972,089 bp). See description for this file under Additional file 5 above. [file 12915_2020_738_MOESM6_ESM.tiff]

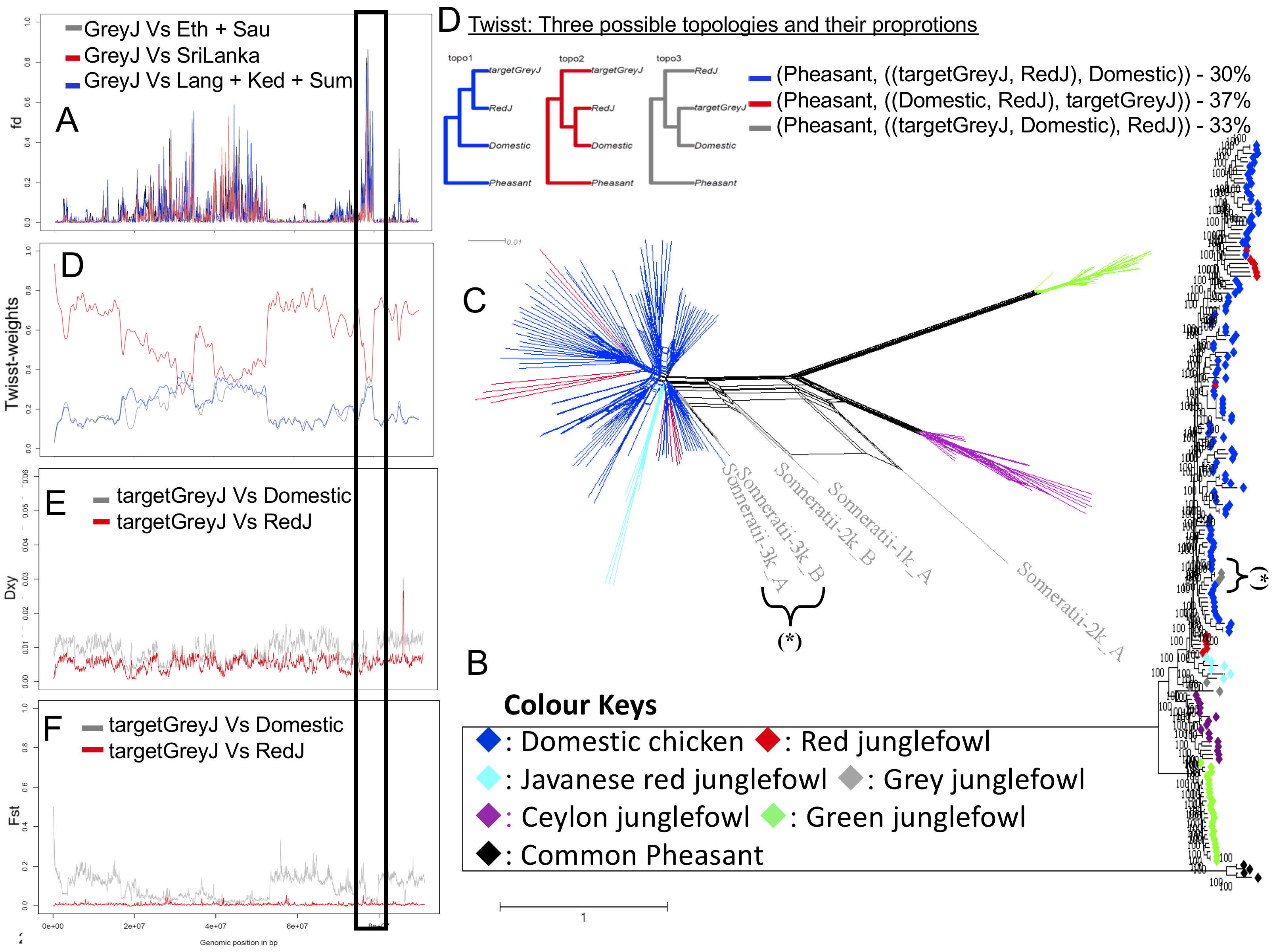

Supplement: Supplementary file 7 — Additional file 7: Figure S5. A 2.8 Mb introgressed region on chromosome 4 (76429662–79,206,239 bp). See description for this file under Additional file 5 above. [file 12915_2020_738_MOESM7_ESM.tiff]

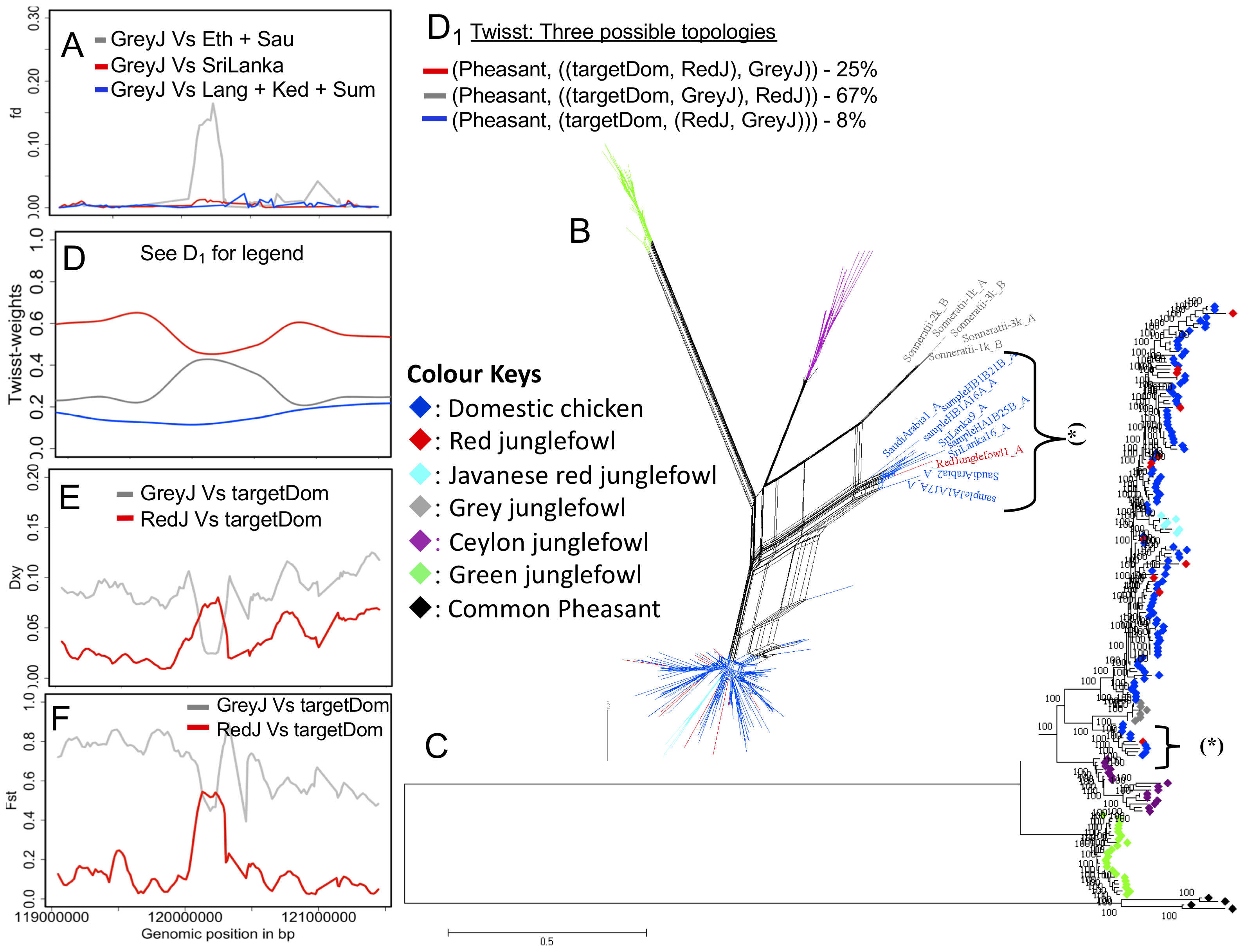

Supplement: Supplementary file 9 — Additional file 9: Figure S6. A 220 kb (Chr 2: 119676880–119,901,132 bp) introgressed region from Grey junglefowl into domestic chicken. targetDom here include the introgressed domestic chicken and a single Red junglefowl haplotypes (*). The following description is applicable to the Additional files 9, 10, 11, 12, 13, 14 and 15 which show figures for the introgressed regions from the Grey junglefowl to domestic chicken/Red junglefowl. The plots are zoomed close to the region. A fd plot, B haplotype-based network and C maximum likelihood tree for the introgressed region. D Twisst plot and D1 its proportion for each of the three possible topologies in the introgressed region. E dXY and F FST. Eth, Sau, SriLanka, Lang, Ked, Sum represent chicken samples from Ethiopia, Saudi Arabia, Sri Lanka, Langshan (China), Kedu Hitam and Sumatra (Indonesia), respectively. GreyJ represent Grey junglefowl, and targetDom are the introgressed (*) domestic haplotypes. [file 12915_2020_738_MOESM9_ESM.tiff]

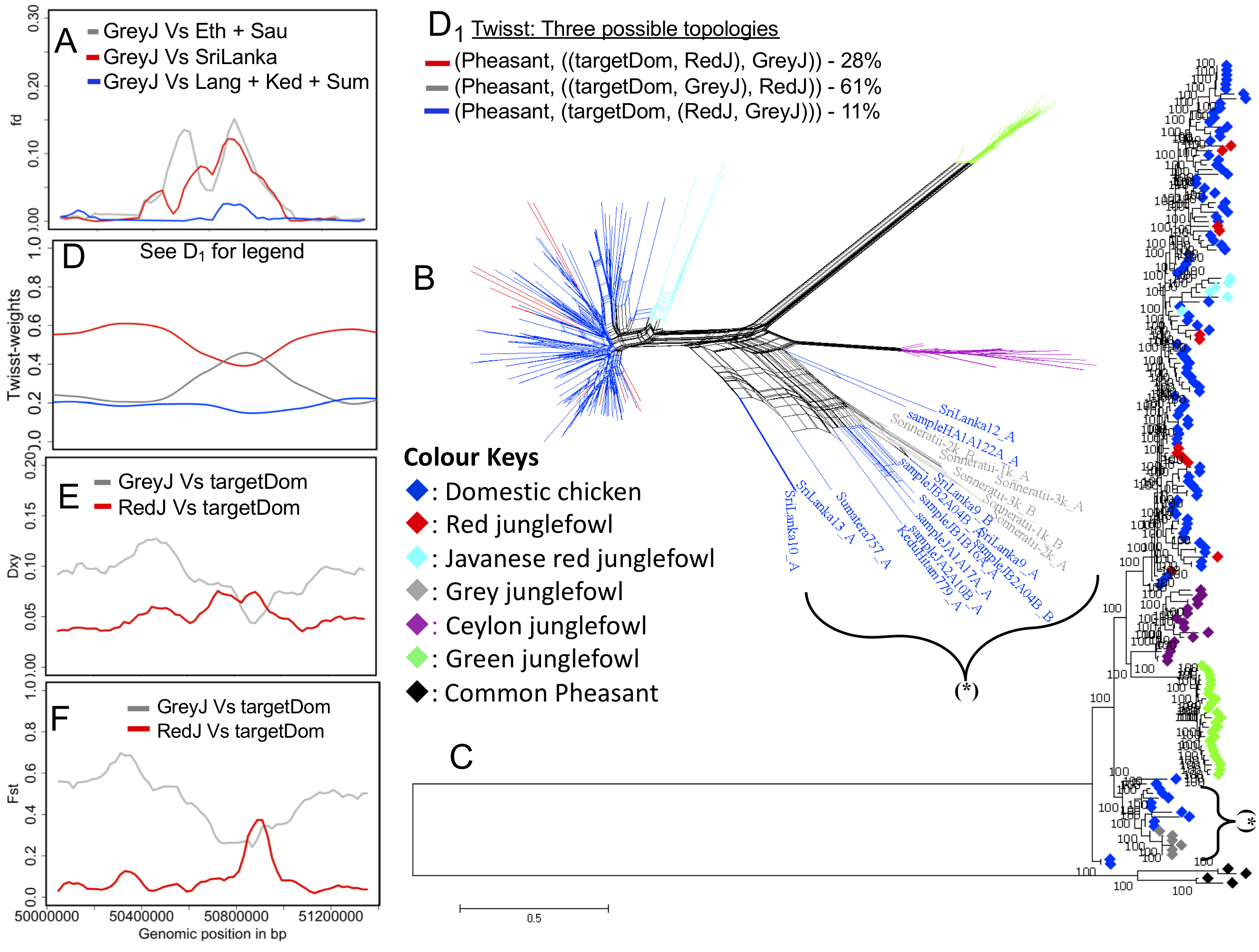

Supplement: Supplementary file 10 — Additional file 10: Figure S7. A 100 kb (Chr 3: 50759656–50,859,645 bp) introgressed region from Grey junglefowl into domestic chicken. See description for this file under Addtional file 9 above. [file 12915_2020_738_MOESM10_ESM.tiff]

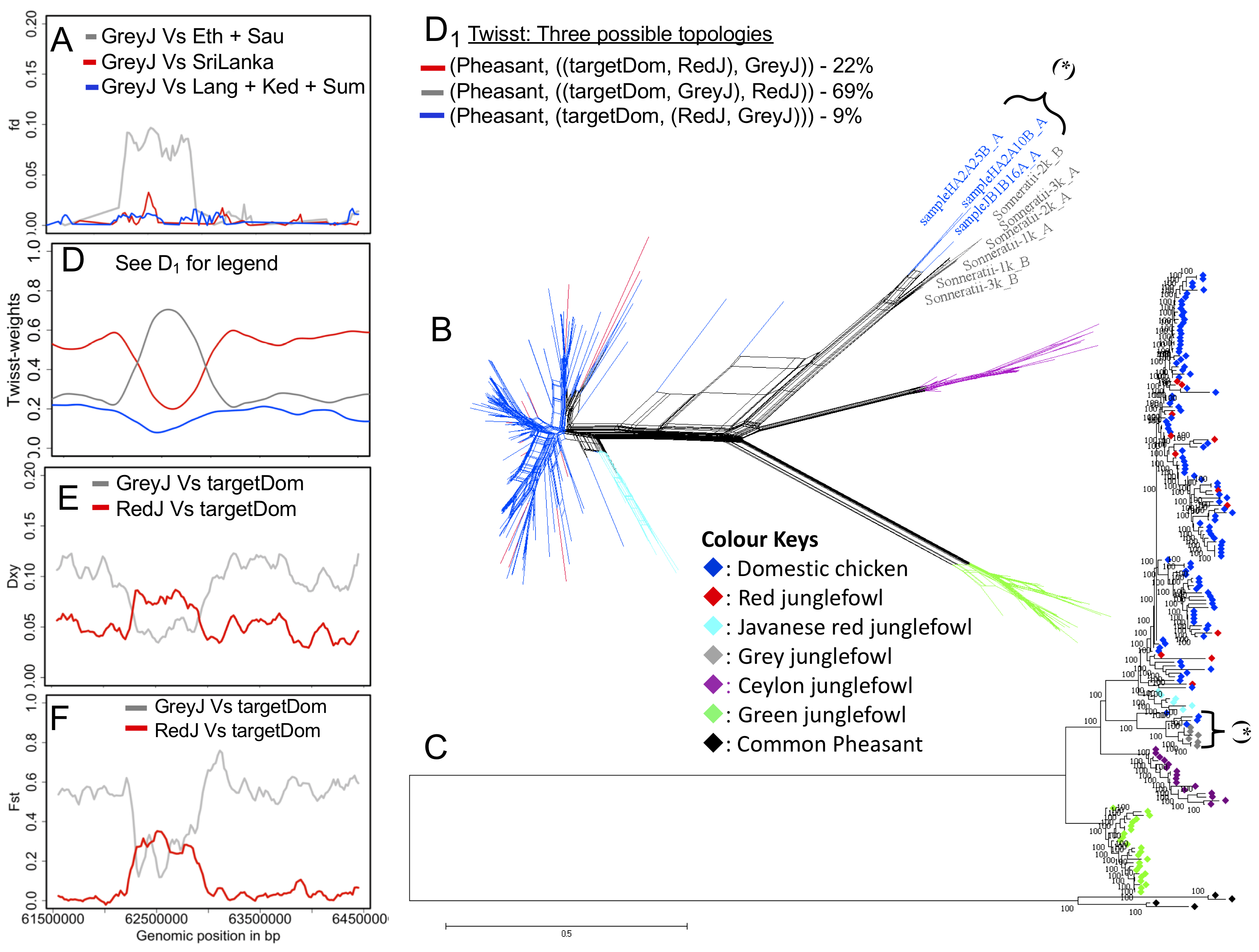

Supplement: Supplementary file 11 — Additional file 11: Figure S8. A 200 kb (Chr 4: 62097304–62,297,319 bp) introgressed region from Grey junglefowl into domestic chicken. See description for this file under Addtional file 9 above. [file 12915_2020_738_MOESM11_ESM.tiff]

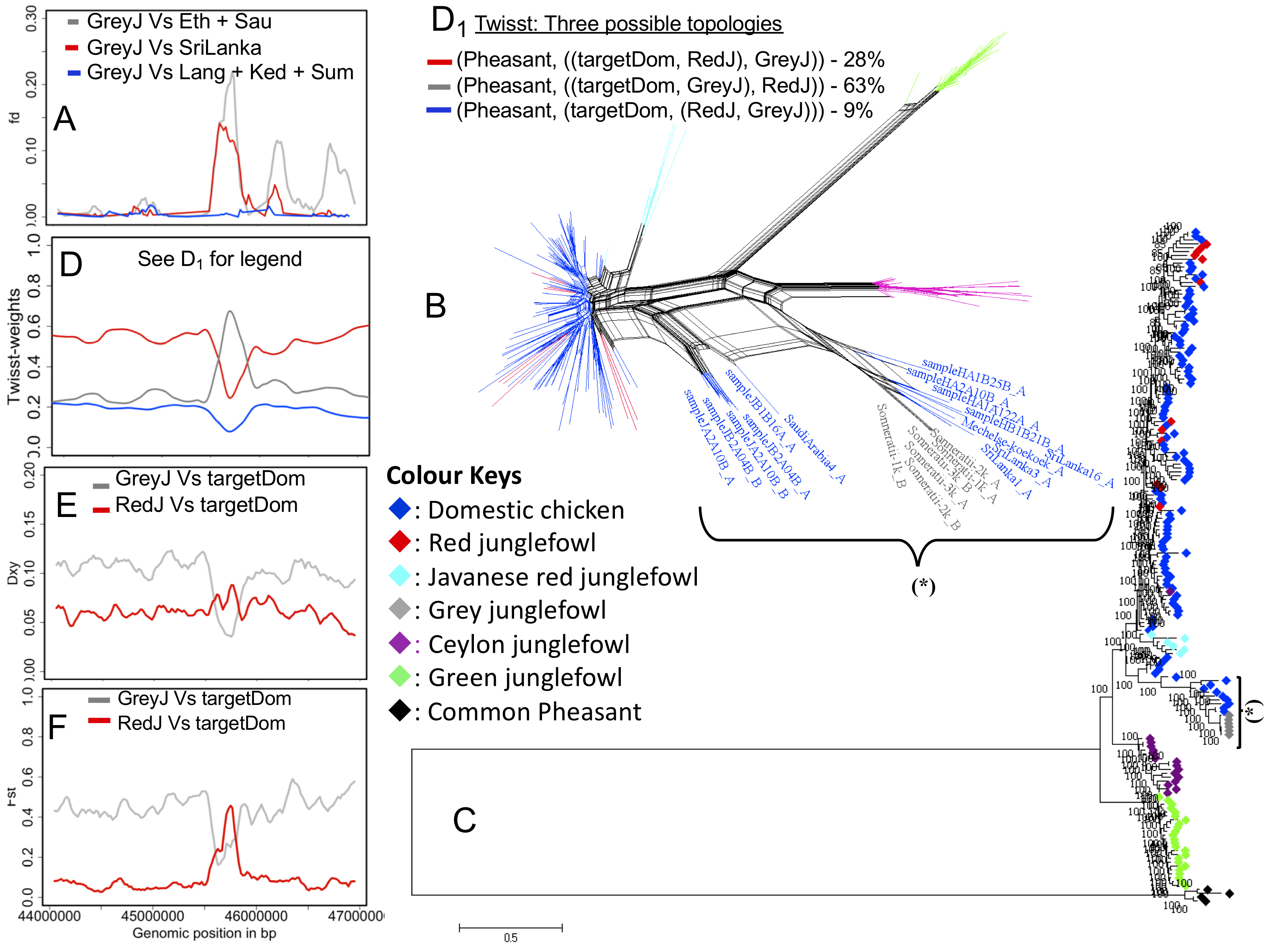

Supplement: Supplementary file 12 — Additional file 12: Figure S9. A 280 kb (Chr 5: 45674368–45,954,418 bp) introgressed region from Grey junglefowl into domestic chicken. See description for this file under Addtional file 9 above. [file 12915_2020_738_MOESM12_ESM.tiff]

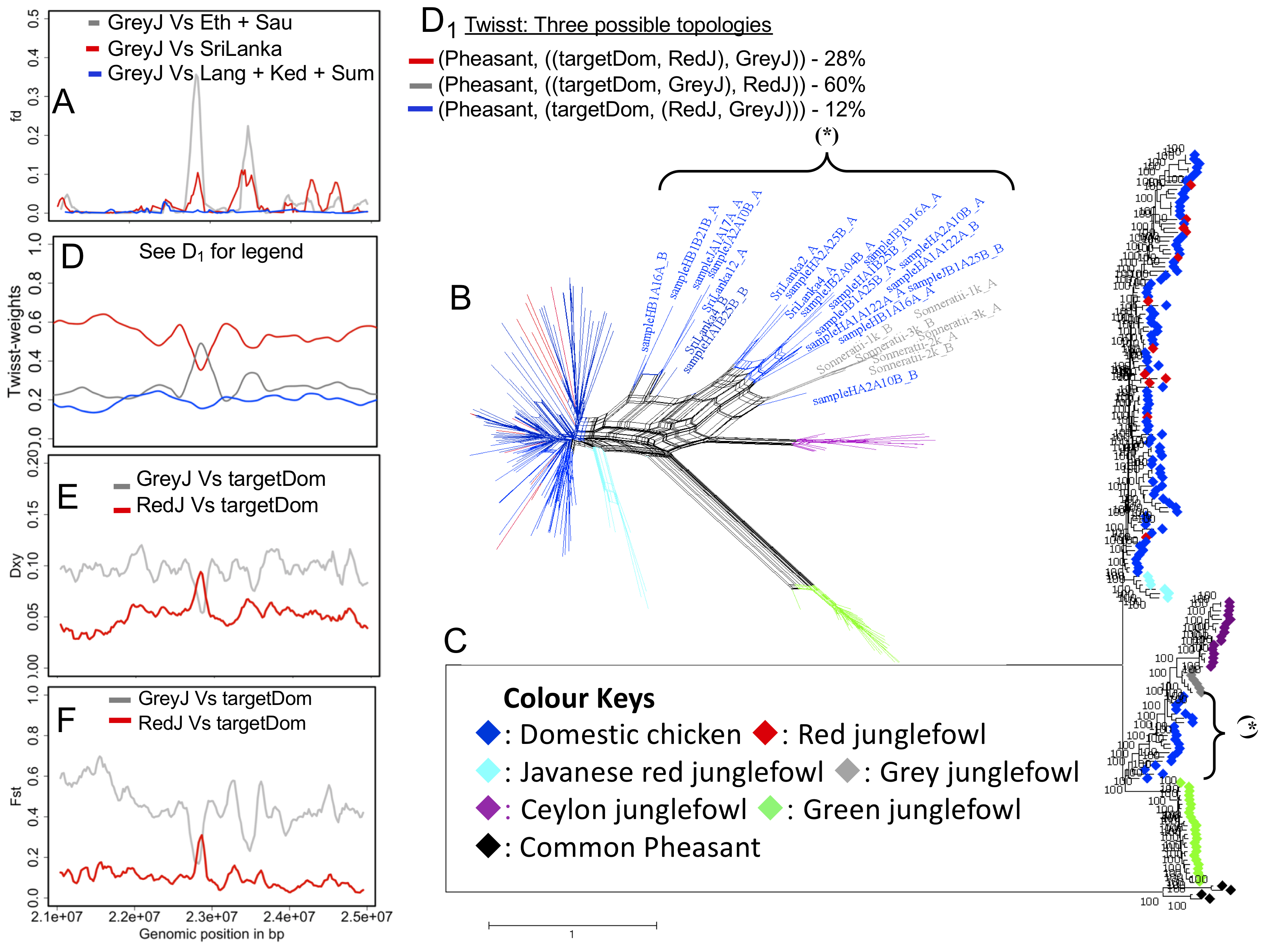

Supplement: Supplementary file 13 — Additional file 13: Figure S10. A 140 kb (Chr 7: 22652767–22,792,759 bp) introgressed region from Grey junglefowl into domestic chicken. See description for this file under Addtional file 9 above. [file 12915_2020_738_MOESM13_ESM.tiff]

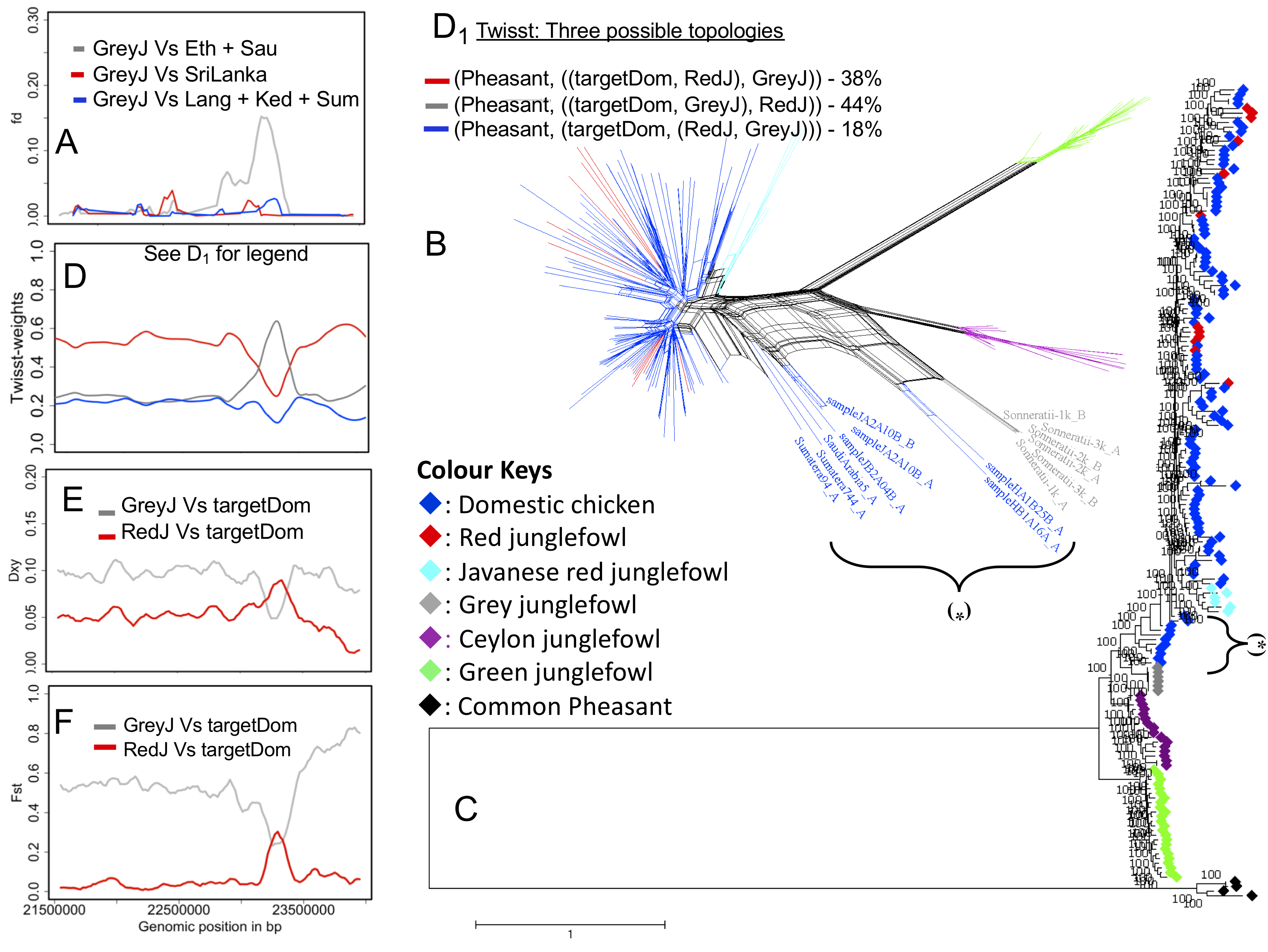

Supplement: Supplementary file 14 — Additional file 14: Figure S11. A 500 kb (Chr 9: 23052049–23,552,045 bp) introgressed region from Grey junglefowl into domestic chicken. See description for this file under Addtional file 9 above. [file 12915_2020_738_MOESM14_ESM.tiff]

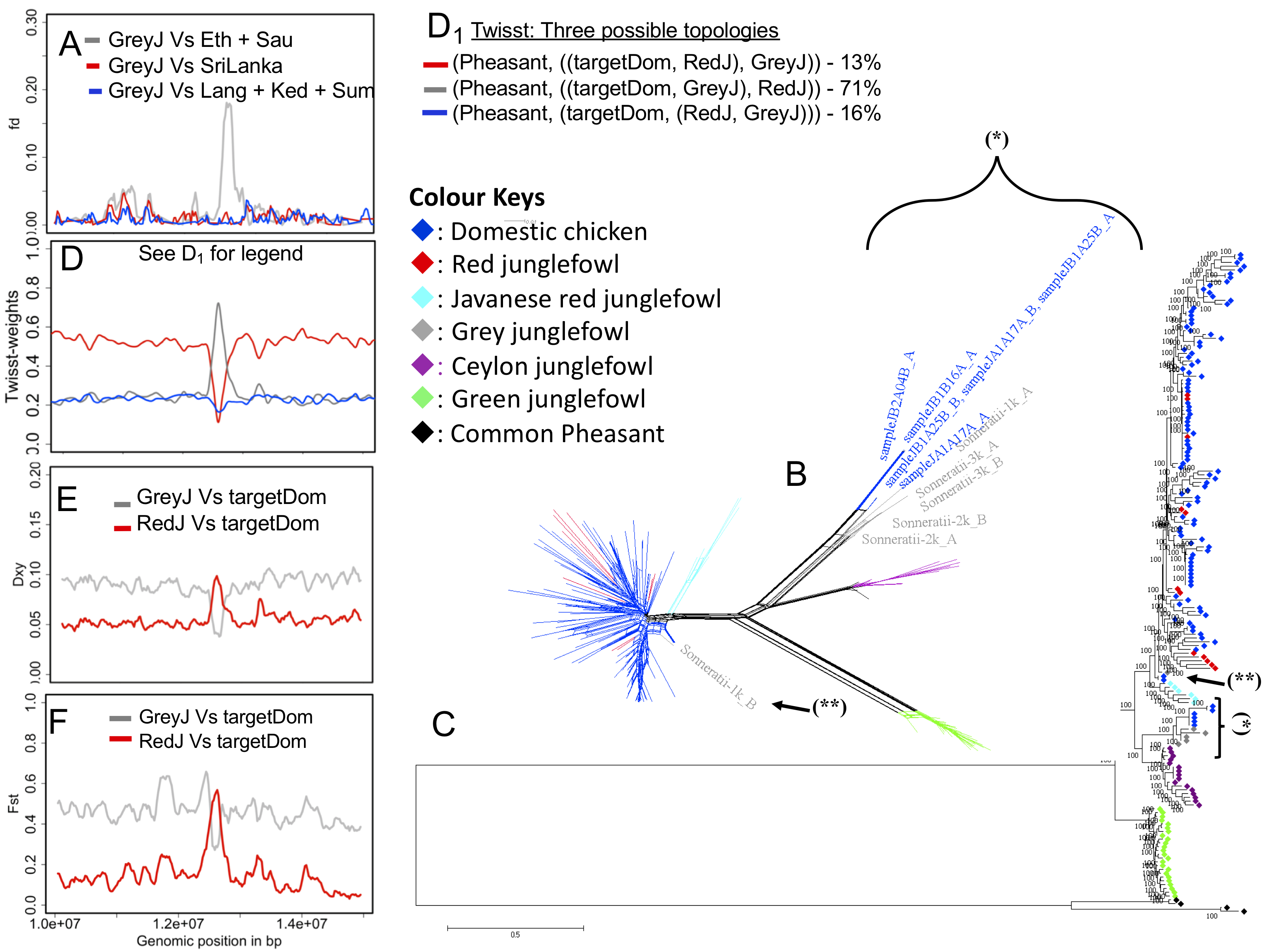

Supplement: Supplementary file 15 — Additional file 15: Figure S12. A 100 kb (Chr 12: 12914268–13,014,266 bp) introgressed region from (*) Grey junglefowl into domestic chicken and (**) from domestic chicken to Grey junglefowl. The Twisst values and plots are based on the introgressed domestic haplotypes from the Grey junglefowl and do not account for the reverse introgression. See description for this file under Addtional file 9 above. [file 12915_2020_738_MOESM15_ESM.tiff]

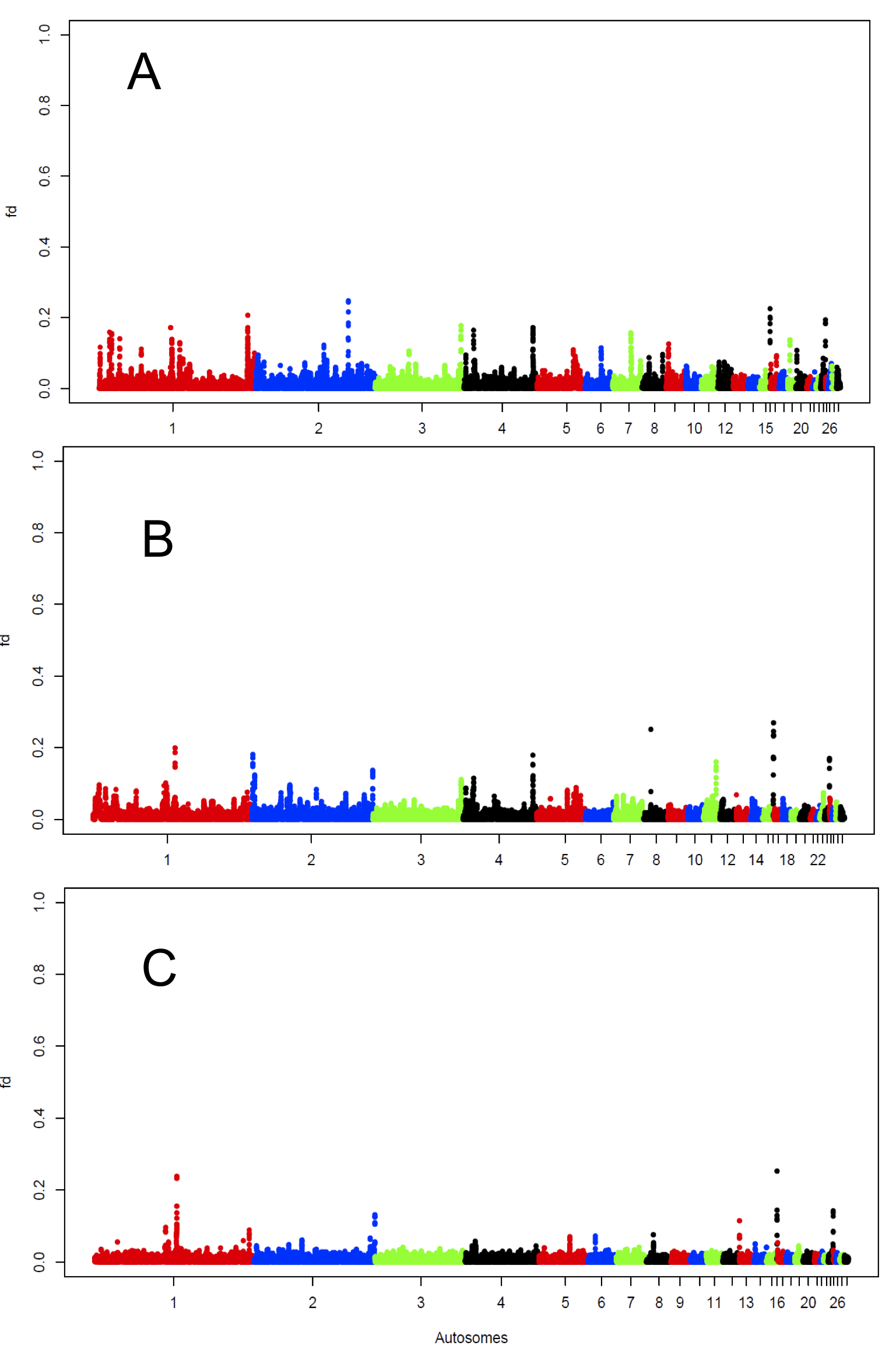

Supplement: Supplementary file 16 — Additional file 16: Figure S13. The fd plots test for the comparison between Ceylon junglefowl and domestic chicken population from (A) Ethiopia and Saudi, (B) Sri Lanka and (C) Southeast and East Asia. The Y-axis fd value and X-axis 1–28 autosomes. [file 12915_2020_738_MOESM16_ESM.tiff]

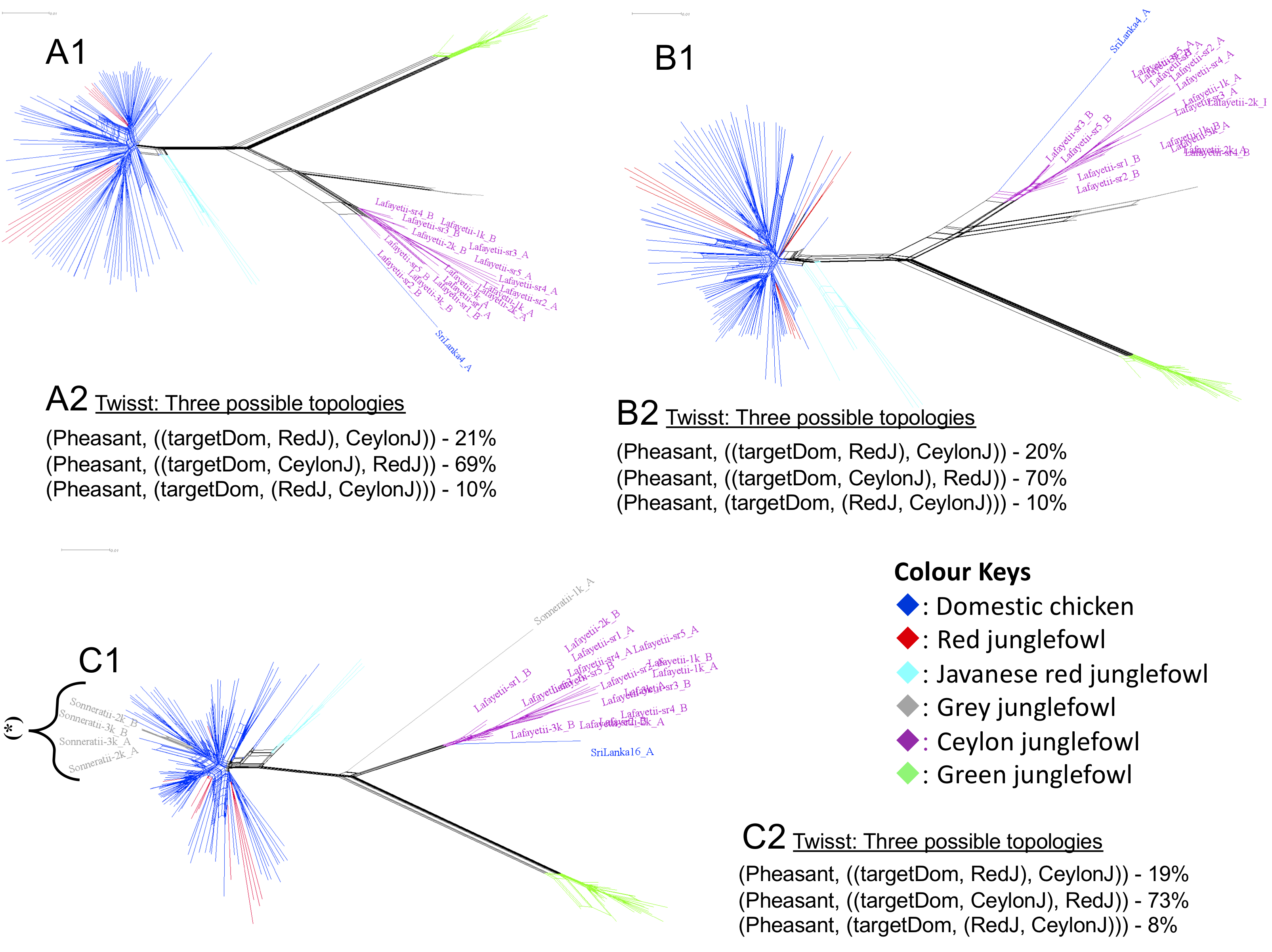

Supplement: Supplementary file 17 — Additional file 17: Figure S14. Network and Twisst proportion of topologies for three Ceylon candidate introgressed regions into domestic chicken (A - C). (A1) and (A2) 6.52 Mb region Chr 1: 2895616–9,418,660 bp, (B1) and (B2) 3.95 Mb Chr 1: 25261354–29,205,161 bp, (C1) and (C2) 1.38 Mb region Chr 1: 147936229–149,316,591 bp. C1 also shows support for (*) introgression from domestic chicken to some Grey junglefowl haplotypes at the same region. [file 12915_2020_738_MOESM17_ESM.tiff]

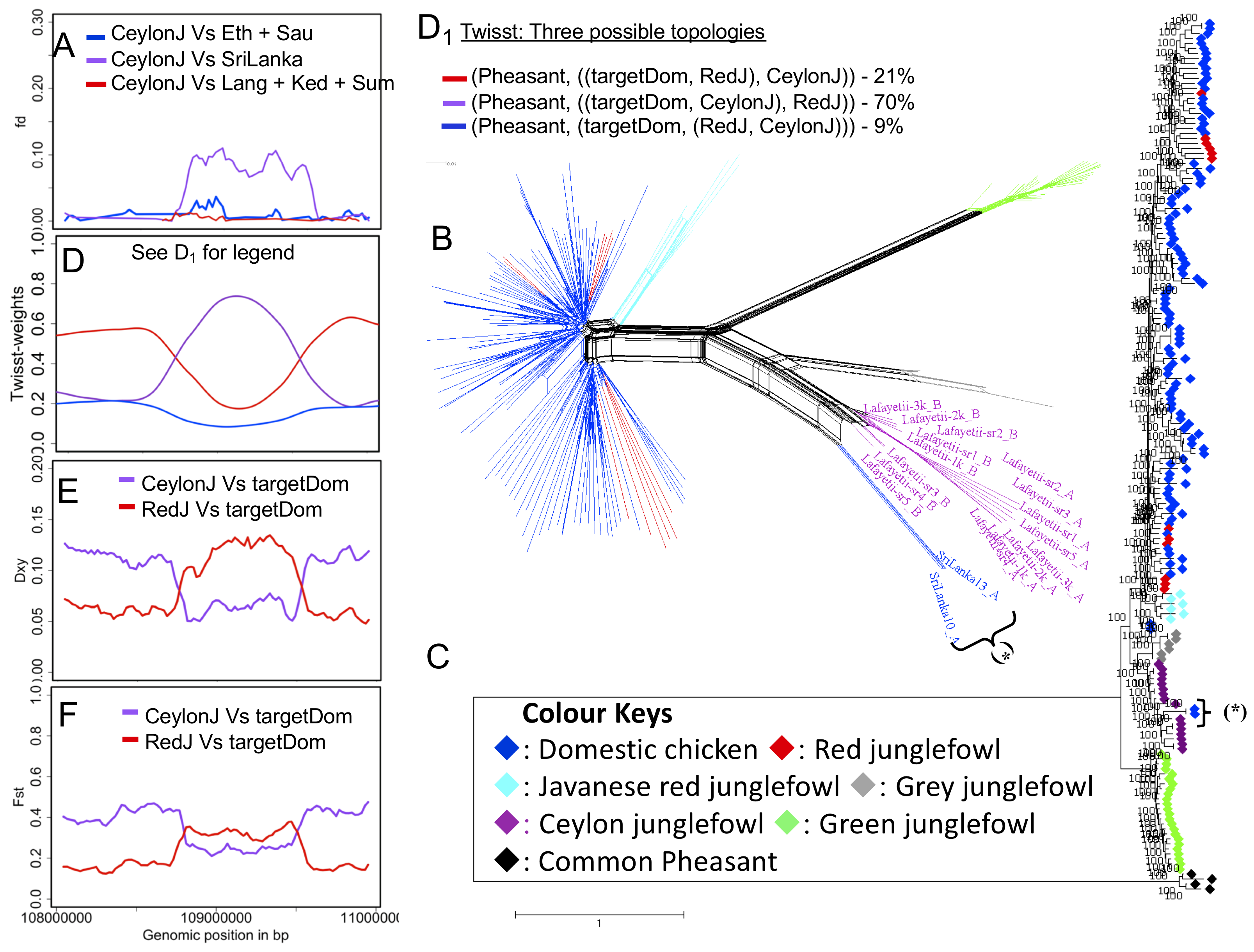

Supplement: Supplementary file 18 — Additional file 18: Figure S15. A 600 kb (Chr 3: 108325801–108,925,723 bp) introgressed region from Ceylon junglefowl to domestic chicken. A fd plot, B haplotype-based network, C maximum likelihood tree, D Twisst plot and D1 its proportion for each of the three possible topologies, E dXY and F FST. Eth, Sau, SriLanka, Lang, Ked, Sum represent chicken samples from Ethiopia, Saudi Arabia, Sri Lanka, Langshan (China), Kedu Hitam and Sumatra (Indonesia), respectively. CeylonJ is Ceylon junglefowl and targetDom are the introgressed domestic chicken haplotypes (*). [file 12915_2020_738_MOESM18_ESM.tiff]

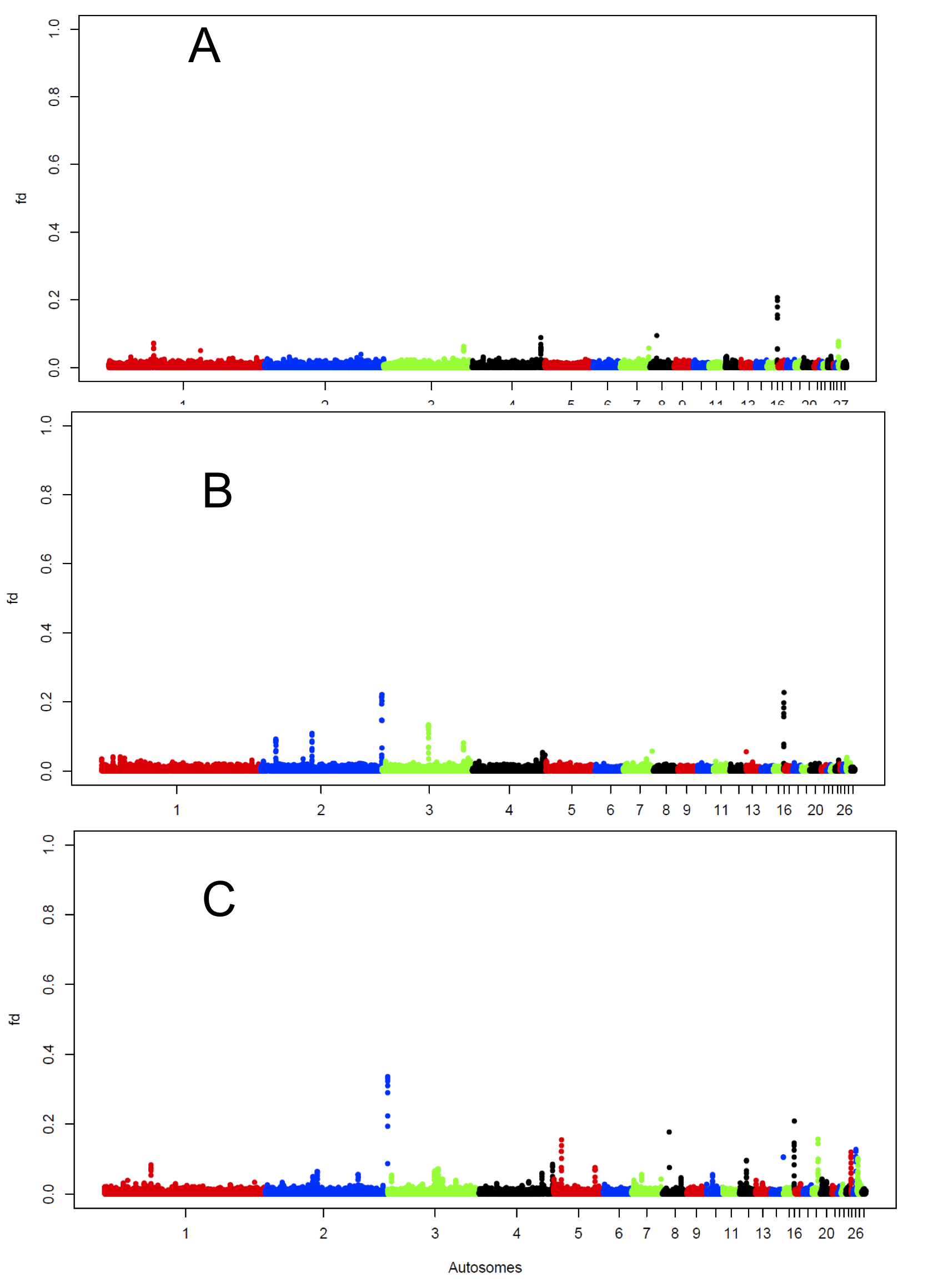

Supplement: Supplementary file 19 — Additional file 19: Figure S16. The fd plots test for the comparison between Green junglefowl and domestic chicken population from (A) Ethiopia and Saudi, (B) Sri Lanka and (C) Southeast and East Asia. The Y-axis fd value and X-axis 1–28 autosomes. [file 12915_2020_738_MOESM19_ESM.tiff]

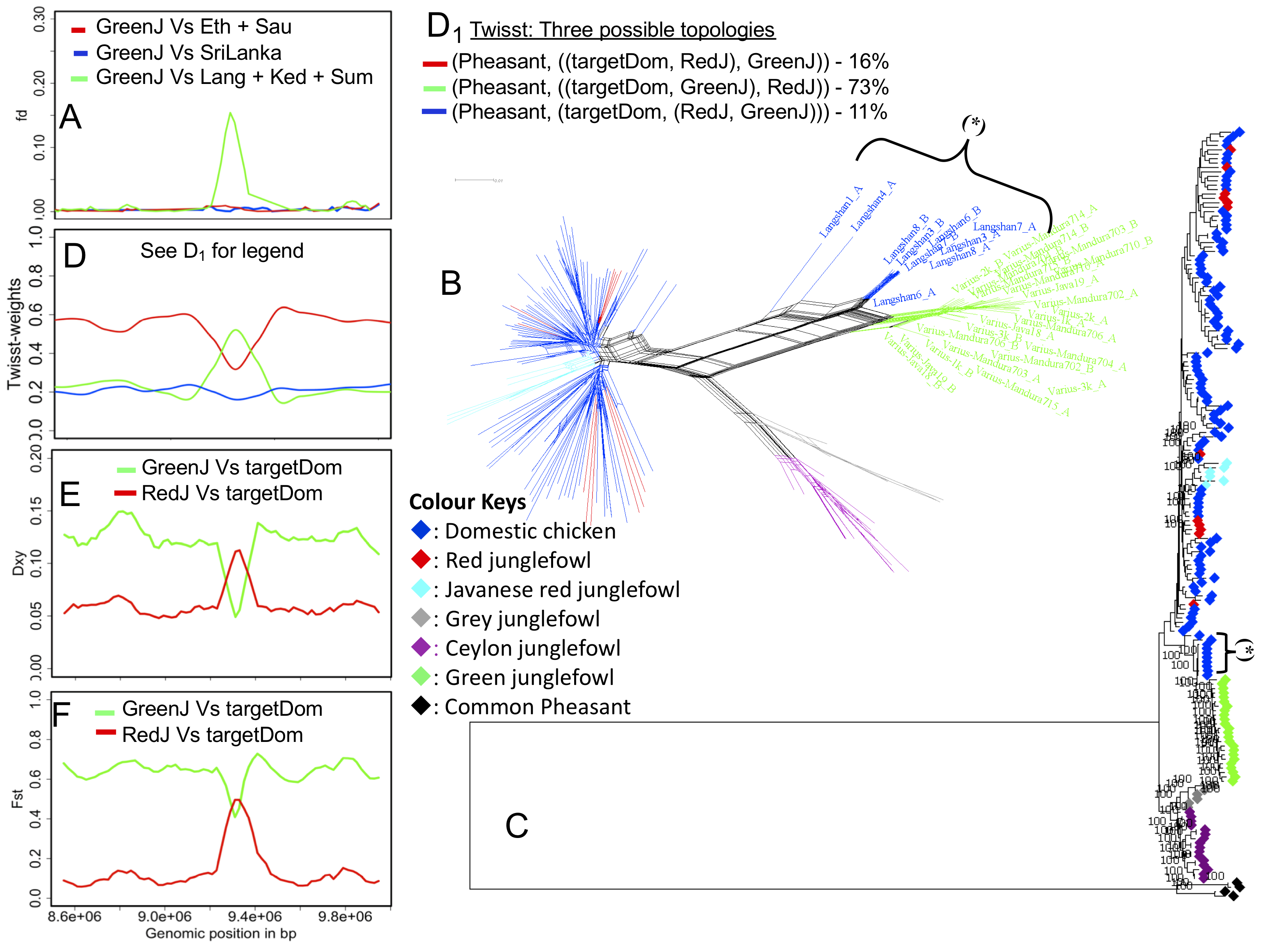

Supplement: Supplementary file 20 — Additional file 20: Figure S17: A 100 kb (Chr 5: 9538715–9,638,713 bp) introgressed region from Green junglefowl into domestic chicken. A fd plot, B haplotype-based network, C maximum likelihood tree, D Twisst plot and D1 its proportion for each of the three possible topologies. E dXY and F FST. Eth, Sau, SriLanka, Lang, Ked, Sum represent chicken samples from Ethiopia, Saudi Arabia, Sri Lanka, Langshan (China), Kedu Hitam and Sumatra (Indonesia), respectively. GreenJ is Green junglefowl and targetDom are the introgressed domestic haplotypes (*). [file 12915_2020_738_MOESM20_ESM.tiff]
