# Supplementary material for: The wild species genome ancestry of domestic chickens
Source: BMC Biol. 2020 Feb 12;18:13. doi: 10.1186/s12915-020-0738-1 (PMC7014787; doi:10.1186/s12915-020-0738-1)
Supplement: Supplementary file 4 — Additional file 4 : Table S2. Candidate introgressed regions from domestic chicken/Red junglefowl into Grey/Ceylon junglefowl. *Positions along the chromosome in megabase (Mb). [file 12915_2020_738_MOESM4_ESM.docx]

**Table S2**. Candidate introgressed regions from domestic chicken/Red junglefowl into Grey/Ceylon junglefowls

| **Candidate introgressed regions*** | **Length** | **Genes within the candidate introgressed regions***** |
| --- | --- | --- |
| ***Domestic chicken/Red junglefowl into Grey Junglefowl*** | |  |
| Chr1: 141.0 – 167.0 | 26 Mb | *TNFSF13B, ABHD13, LIG4, FAM155A, ARGLU1, EFNB2, SLC10A2, ERCC5, BIVM, KDELC1, TEX30, METTL21C, TPP2, FHF-4, ITGBL1, NALCN, TMTC4, GGACT, PCCA, ZIC2, ZIC5, CLYBL, TM9SF2, gga-mir-2984, UBAC2, GPR183, GPR18, DOCK9, SLC15A1, STK24, FARP1, gga-mir-1555, IPO5, RAP2A, MBNL2, UGGT2, DNAJC3, DZIP1, CLDN10, ABCC4, SOX21, GPR180, TGDS, DCT, RF00066, GPC5, gga-mir-92-1, gga-mir-19b, gga-mir-20a, gga-mir-19a, gga-mir-18a, gga-mir-17, SLITRK6, SLITRK1, SPRY2, NDFIP2, RBM26, RNF219, POU4F1, EDNRB, SLAIN1, MYCBP2, FBXL3, CLN5, GATD3A, ACOD1, KCTD12, LMO7, UCHL3, TBC1D4, KLF12, KLF5, PIBF1, DIS3, BORA, MZT1, DACH1, gga-mir-1743, KLHL1, PCDH9, RF00154, gga-mir-7445-2, TDRD3, DIAPH3, PCDH17, RF02271, RF00493, RF00494, OLFM4, PCDH8, CNMD, SUGT1, ELF1, WBP4, MTRF1, RGCC, VWA8* |
| Chr2: 11.0 – 20.0 | 9 Mb | *PFKP, PITRM1, KLF6, gga-mir-6628, GJD4, CCNY, CREM, CUL2, PARD3, RF02271, NRP1, ITGB1, EPC1, gga-mir-1768, KIF5B, ARHGAP12, ZEB1, ZNF438, SVIL, JCAD, MTPAP, MAP3K8, BAMBI, WAC, MPP7, ARMC4, MKX, RAB18, YME1L1, MASTL, ACBD5, ABI1, PDSS1, APBB1IP, GAD2, MYO3A, GPR158, THNSL1, ENKUR, PRTFDC1, ARHGAP21, KIAA1217, PTF1A, ARMC3, PIP4K2A, SPAG6, gga-mir-12240, BMI1, COMMD3, DNAJC1, MLLT10, RF00001, NEBL, PLXDC2, MALRD1, ARL5B, NSUN6, CACNB2, SLC39A12, MMR1L2, MMR1L1, MMR1L3, MMR1L4, MRC1, STAM, HACD1, VIM, ST8SIA6, gga-mir-1661, TRDMT1, CUBN* |
| Chr4:76.4 – 79.2 | 2.8 Mb | *TAPT1, Prom1, FGFBP2, CD38, BST1, FBXL5, CC2D2A, C1QTNF7, CPEB2, NKX3-2, RAB28, HS3ST1, ZNF518B, WDR1, SLC2A9, DRD5, OTOP1, TMEM128, LYAR, ZBTB49, NSG1, STX18, MSX1, CYTL1, STK32B, EVC2, EVC, CRMP1B* |
| ***Domestic chicken into Ceylon junglefowl*** | |  |
| Chr5: 49.33 – 49.43 | 100 kb | *No gene* |

*Positions along the chromosome in megabase (Mb)
