# Supplementary material for: The wild species genome ancestry of domestic chickens
Source: BMC Biol. 2020 Feb 12;18:13. doi: 10.1186/s12915-020-0738-1 (PMC7014787; doi:10.1186/s12915-020-0738-1)
Supplement: Supplementary file 8 — Additional file 8: Table S3. Candidate introgressed regions from non-red junglefowl into domestic chicken/Red junglefowl. *Positions along the chromosome in megabase (Mb), **SEA (Southeast and East Asia) (see methods for sampling location), ***Ensembl release version 96. [file 12915_2020_738_MOESM8_ESM.docx]

**Table S3**. Candidate introgressed regions from non-red junglefowls into domestic chicken/Red junglefowl

| **Candidate introgressed regions*** | **Length** | **Proportion of haplotypes introgressed in each population (%)**** | | | | | | | **Genes within the candidate introgressed regions***** |
| --- | --- | --- | --- | --- | --- | --- | --- | --- | --- |
|  |  | Ethiopia  (n = 22) | Saudi Arabia  (n = 10) | Sri Lanka  (n = 22) | SEA Langshan (n = 16) | SEA  Kedu Hitam (n = 20) | SEA Sumatra (n = 10) | Red Junglefowl  (n = 12) |  |
| ***Introgressed regions from Grey junglefowl to domestic chicken/Red junglefowl*** | | | | | | | | | |
| Chr2: 119.68 – 119.90 | 220 kb | 23 | 20 | 9 | 0 | 0 | 0 | 8 | *-* |
| Chr3: 50.75 – 50.85 | 100 kb | 27 | 0 | 23 | 0 | 5 | 10 | 0 | *NOX3* |
| Chr4: 62.10 – 62.30 | 200 kb | 14 | 0 | 0 | 0 | 0 | 0 | 0 | *RF00003* |
| Chr5: 45.67 – 45.95 | 280 kb | 41 | 10 | 9 | 0 | 0 | 0 | 0 | *PPP4R4, SERPINA10, SPIA4, SPIA1, GSC* |
| Chr6: 21.73 – 21.85 | 120 kb | 45 | 0 | 0 | 0 | 0 | 0 | 0 | *IDE, Mar-05, CPEB3* |
| Chr7: 22.65 – 22.79 | 140 kb | 50 | 0 | 9 | 0 | 0 | 0 | 0 | *-* |
| Chr9: 23.05 – 23.55 | 500 kb | 23 | 10 |  | 0 | 0 | 20 | 0 | *KCNAB3, GMPS, gga-mir-1658, C3orf33, PLCH1, MME, GPR149, DHX36, RAP2B, ARHGEF26, P2RY1, MBNL1* |
| Chr12: 12.91 – 13.01 | 100 kb | 27 | 0 | 0 | 0 | 0 | 0 | 0 | *FHIT* |
| ***Introgressed regions from Ceylon junglefowl to domestic chicken*** | | | | | | | | | |
| Chr1: 2.90 – 9.42 | 6.52 Mb | 0 | 0 | 5 | 0 | 0 | 0 | 0 | *PLXNA4, gga-mir-6621, PODXL, MKLN1, gga-mir-29b-1, gga-mir-29a, K123, IL2RA, RBM17, PFKFB3, SFMBT2, ITIH5, ITIH2, KIN, ATP5F1C, TAF3, GATA3, CELF2, gga-mir-1626, gga-mir-1596, USP6NL, ECHDC3, UPF2, DHTKD1, SEC61A2, NUDT5, CDC123, CAMK1D, CCDC3, OPTN, MCM10, PHYH, SEPHS2L, BEND7, FRMD4A, gga-mir-1460, FAM107B, HSPA14, SUV39H2, DCLRE1C, MEIG1, TMEM243, DMTF1, RF02271, KIAA1324L, GRM3, SEMA3D, SEMA3A* |
| Chr1:25.25 – 29.21 | 3.95 Mb | 0 | 0 | 5 | 0 | 0 | 0 | 0 | *TES, TFEC, MDFIC, FOXP2, PPP1R3A, GPR85, gga-mir-1695, BMT2, TMEM168, LSMEM1, IFRD1, ZNF277, DOCK4, IMMP2L, LRRN3, DNAJB9, THAP5, AVPR2, PNPLA8, NRCAM, gga-mir-12208, CNTN1, PDZRN4* |
| Chr1:147.94 – 149.32 | 1.38 Mb | 0 | 0 | 5 | 0 | 0 | 0 | 0 | *GPC6, RF00066, GPC5* |
| Chr3:108.33 – 108.93 | 600 kb | 0 | 0 | 9 | 0 | 0 | 0 | 0 | *CRISP3, CRISP2, RHAG, CYP2AC1, CYP2AC2, CENPQ, MMUT, OPN5L2, FOXP2* |
| ***Introgressed region from Green junglefowl to domestic chicken*** | | | | | | | | | |
| Chr5: 9.54 – 9.64 | 100 kb | 0 | 0 | 0 | 63 | 0 | 0 | 0 | *SWAP70, WEE1, ZNF143, IPO7, RF00319* |

*Positions along the chromosome in megabase (Mb), ******SEA (South-East and East Asia), ***Ensembl release version 96
